# Supplementary material for: Bias-free driven ion assisted photoelectrochemical system for sustainable wastewater treatment
Source: Nat Commun. 2023 Dec 18;14:8413. doi: 10.1038/s41467-023-44155-5 (PMC10728197; doi:10.1038/s41467-023-44155-5)
Supplement: Supplementary file 1 — Supplementary Information [file 41467_2023_44155_MOESM1_ESM.pdf]

Supplementary Information

Bias-Free Driven Ion- Assisted Photoelectrochemical System for Sustainable Wastewater Treatment

Qi Dang [1,6], Wei Zhang[2,6], Jiqing Liu[1], Liting Wang[1], Deli Wu[3], Dejin Wang[5], Zhendong Lei[3,4]*, and Liang Tang[1,5]*

[1]Key Laboratory of Organic Compound Pollution Control Engineering (MOE), School of Environmental and Chemical Engineering, Shanghai University, Shanghai 200444, China

[2]Department of Chemistry, IRIS Adlershof & The Center for the Science of Materials Berlin, Humboldt-Universität zu Berlin, Brook-Taylor-Str. 2, 12489 Berlin, Germany

[3]College of Environmental & Engineering, Tongji University, Shanghai 200092, China

[4]School of Materials Science and Engineering, Nanyang Technological University, Singapore 639798, Singapore

[5]School of Resources and Environment, Anqing Normal University, Anqing 246011, China

[6]These authors contributed equally: Qi Dang, Wei Zhang.

*E-mail: leizd95@tongji.edu.cn (Z.L); tang1liang@shu.edu.cn(L,T)

Contents

Supplementary Figure 1. Structural characterization of TiO_2 photoanode: (a) SEM image and (b) XRD spectra of TiO_2 film grown on a FTO conductive substrate. 7

Supplementary Figure 2. Structural characteristics of FeFe Prussian blue: (a) SEM image and (b) XRD pattern. 8

Supplementary Figure 3. Structural characteristics of CoFe Prussian blue analogues (PBAs): (a) SEM image and (b) XRD pattern. 9

Supplementary Figure 4. Structural characteristics of NiFe Prussian blue analogues: (a) SEM image and (b) XRD pattern. 10

Supplementary Figure 5. Structural characteristics of CuFe Prussian blue analogues: (a) SEM image and (b) XRD pattern. 11

Supplementary Figure 6. Structural characterization of $BiVO_4$ photoanode: (a) Ultraviolet-visible absorption spectrum of TiO_2 and $BiVO_4$ film and (b) XRD spectrum of $BiVO_4$ film grown on a FTO conductive glass substrate. 12

Supplementary Figure 7. Our IAPEC test unit (a)left view and (b) front view. 13

Supplementary Figure 8. Distribution of the irradiation intensity of the simulated sunlight. 14

Supplementary Figure 9. Open circuit voltage (OPV) of different PEC systems. 15

Supplementary Figure 10. The theoretical calculation of the Gibbs free energy change for the FeFe PB structure during the insertion process of Na^+, K^+ $NH_4^+$and H^+ ions. 16

Supplementary Figure 11. The theoretical calculation of the Gibbs free energy change for the NiFe PBA structure during the insertion process of Na^+, K^+ $NH_4^+$and H^+ ions. 17

Supplementary Figure 12. The theoretical calculation of the Gibbs free energy change for the CoFe PBA structure during the insertion process of Na^+, K^+ $NH_4^+$and H^+ ions............ 18

Supplementary Figure 13. The Na *1s* XPS spectra of CuFe PBA electrode before and after ion insertion in the IAPEC system. 19

Supplementary Figure 14. High-resolution XPS spectra of Fe 2*p* in CuFe PBA before and after interaction with Na^+ ions. 20

Supplementary Figure 15. (a) XRD patterns of CuFe PBA electrode before and after ion insertion in the IAPEC system (b) Enlarged view of the (200) crystal plane. (c) Enlarged view of the (220) crystal plane. (d) Enlarged view of the (400) crystal plane. 21

Supplementary Figure 16. (a) Schematic structure CuFe PBA sample before and (b) after Na^+ ions insertion. (c) Rietveld refinement synchrotron PXRD pattern of CuFe PBA sample before and (d) after Na^+ ions insertion. 22

Supplementary Figure 17. Raman spectra of CuFe PBA sample before and after Na^+ ions insertion. 23

Supplementary Figure 18. The degradation pseudo-first-order rate constant of IAPEC for MB by different system. 24

Supplementary Figure 19. Transient photocurrent densities of PEC and IAPEC systems under intermittent simulated sunlight irradiation in different concentrations 25

Supplementary Figure 20. Degradation of MB in different systems and concentrations 26

Supplementary Figure 21. Degradation of IBP in different systems and pollution concentrations 27

Supplementary Figure 22. Degradation of CBZ in different systems and concentrations .. 28

Supplementary Figure 23. The degradations performances of PEC (a) and IAPEC (b) systems for Bisphenol A (BPA), 4-Chlorophenol (4-CP), Perfluorooctanoic Acid (PFOA), Sulfamethoxazole (SMX) and Cellulose Acetate Propionate (CAP). 29

Supplementary Figure 24. The removal rate of TOC of eight model pollutants by IAPEC system after 2 h reaction. 30

Supplementary Figure 25. Concentrations of toxic oxychlorides during the IAPEC treatment of saline sewage with different Photoanode. 31

Supplementary Figure 26. Reusability performance of MB after 20 cycles 32

Supplementary Figure 27. Reusability performance of IBP after 20 cycles 33

Supplementary Figure 28. Reusability performance of CBZ after 20 cycles....................... 34

Supplementary Figure 29. X-ray diffraction patterns of CuFe PBA electrode after 20 cycles. 35

Supplementary Figure 30. The influence of pH variations on the MB degradation performance of the IAPEC system. 36

Supplementary Figure 31. The generation of free chlorine (FCS: Cl_2, HClO/ClO) during MB degradation in the IAPEC system... 37

Supplementary Figure 32. The influence of temperature variations on the degradation performance of the IAPEC system. 38

Supplementary Figure 33. The influence of NaCl, KCl, $CaCl_2$ and $MgCl_2$ on the degradation performance of the IAPEC system. 39

Supplementary Figure 34. The transient photocurrent density recorded for IAPEC system with and without sunlight irradiation in different electrolyte of 0.1 M NaCl, 0.1 M KCl, 0.05 M $CaCl_2$, 0.05 M $MgCl_2$. simulated solar light illumination 100 mW cm^{-2} xenon lamp source at 30 s intervals throughout a 270 s running. 40

Supplementary Figure 35. (a) Degradation effect of different salts on MB and (b) pseudo-first-order rate constant 41

Supplementary Figure 36. The influence of the effects of humic acid (HA) on the degradation performance of the IAPEC system. 42

Supplementary Figure 37. The different free radical scavengers quenched. 43

Supplementary Figure 38. ESR spectra of •Cl, and •Cl_2 in the PEC system with DMPO as a spin trap. 44

Supplementary Figure 39. ESR spectra of DMPO-·OH and DMPO-·SO_4^- adducts under sulfate media in IAPEC system. 45

Supplementary Figure 40. ESR spectra of DMPO-·OH and DMPO-·SO_4^- adducts in sulfate media with 10 ppm IBP in IAPEC system. 46

Supplementary Figure 41. ESR spectra of TEMP-h^+ adduct in sulfate media in our IAPEC system. 47

Supplementary Figure 42. The effect of quenchers on MB (10 ppm) degradation and the pseudo-first-order rate constants for quenching experiments in sulfate media in IAPEC system 48

Supplementary Figure 43. Corresponding first-order kinetic curves of probe experiments for IAPEC system in sulfate media. 49

Supplementary Figure 44. Comparison of contributions of active species calculated based on quenching and probe experiments (a) IAPEC under sulphate medium. (b) IAPEC under chloride medium. 50

Supplementary Figure 45. Fukui function configuration analysis of $C_{12}H_{10}N_3S^+$ molecular configuration after optimization (with numbering). 51

Supplementary Figure 46. Isosurface diagram of Fukui function of $C_{12}H_{10}N_3S^+$ molecule: (a) Electrophilic offense index f^+, (b) Nucleophilic aggression index f^-, (c) Free radical attack index f^0. 52

Supplementary Figure 47. Isosurface diagram of Fukui function of $C_6H_6NO_2^+$ molecular configuration after optimization (with numbering). 53

Supplementary Figure 48. Isosurface diagram of Fukui function of $C_6H_6NO_2^+$ molecule: (a) Electrophilic offense index f^-, (b) Nucleophilic aggression index f^+, (c) Free radical attack index f^0. 54

Supplementary Figure 49. Raw data of LCMS. 56

Supplementary Figure 50. A schematic diagram for the experimental conditions of IAPEC, PC, EC and PEC. 57

Supplementary Table 1. Fe pre-edge peak analysis on ex situ samples and the average oxidation state of Fe species in samples. 58

Supplementary Table 2. EXAFS fitting parameters at the Fe K-edge for various samples. 59

Supplementary Table 3. Comparison of the photoelectrochemical performance of various PEC-Cl. 60

Supplementary Table 4. The pH changes of simulated saline wastewater before and after degradation with different initial pH values. 61

Supplementary Table 5. Hirshfeld charges and calculated f^-, f^+, f^0 and CDD of $C_{12}H_{10}N_3S^+$. 62

Supplementary Table 6. Hirshfeld charges and calculated f^-, f^+, f^0 and CDD of $C_6H_6NO_2^+$. 63

Supplementary Figures

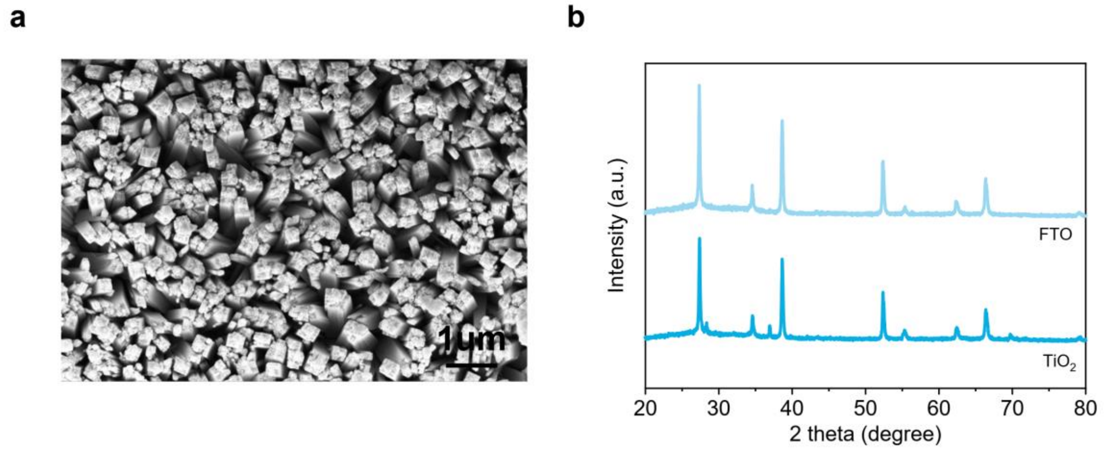

Supplementary Figure 1. Structural characterization of TiO_2 photoanode: (a) SEM image and (b) XRD spectra of TiO_2 film grown on a FTO conductive substrate.

The SEM image and XRD spectra indicate that the synthesized titanium dioxide film is of the anatase phase, exhibiting a nanotube morphology.

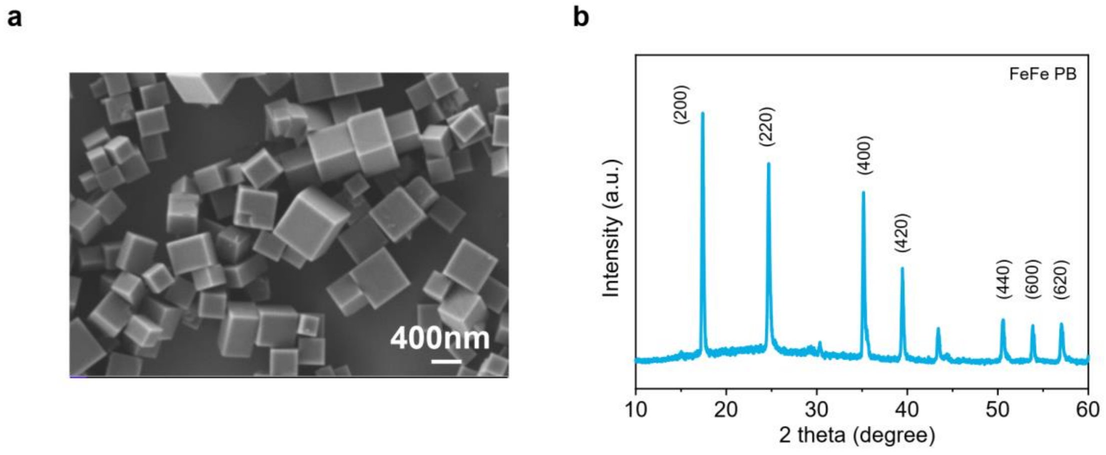

Supplementary Figure 2. Structural characteristics of FeFe Prussian blue: (a) SEM image and (b) XRD pattern.

The SEM image and XRD spectrum indicate that the synthesized FeFe Prussian blue is of the cubic phase, exhibiting a cube morphology.

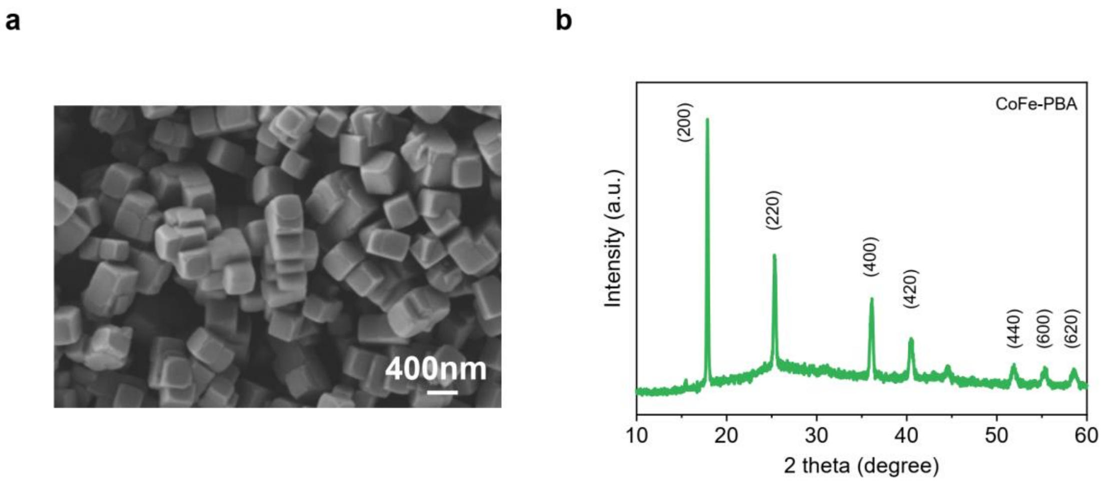

Supplementary Figure 3. Structural characteristics of CoFe Prussian blue analogues (PBAs): (a) SEM image and (b) XRD pattern.

The SEM image and XRD pattern show that the synthesized CoFe PBA is of the cubic phase, exhibiting a cube morphology.

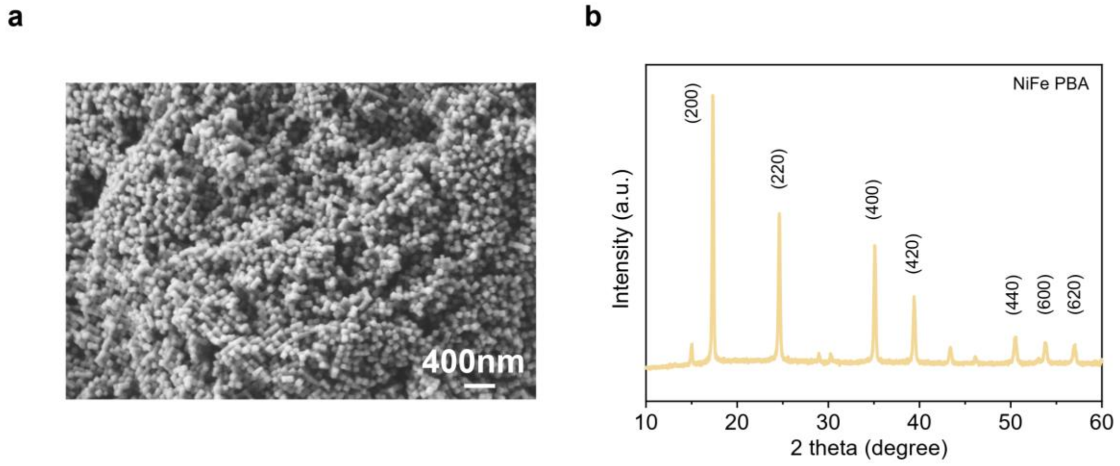

Supplementary Figure 4. Structural characteristics of NiFe Prussian blue analogues: (a) SEM image and (b) XRD pattern.

The SEM image and XRD pattern show that the synthesized NiFe PBA is of the cubic phase, exhibiting a cube morphology.

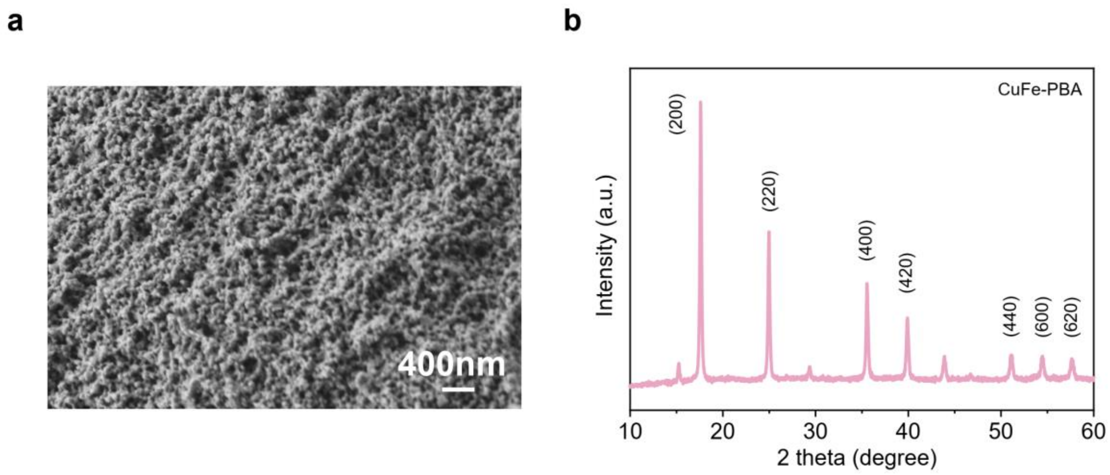

Supplementary Figure 5. Structural characteristics of CuFe Prussian blue analogues: (a) SEM image and (b) XRD pattern.

The SEM image and XRD pattern show that the synthesized CuFe PBAs is of the cubic phase, exhibiting an irregular particle morphology.

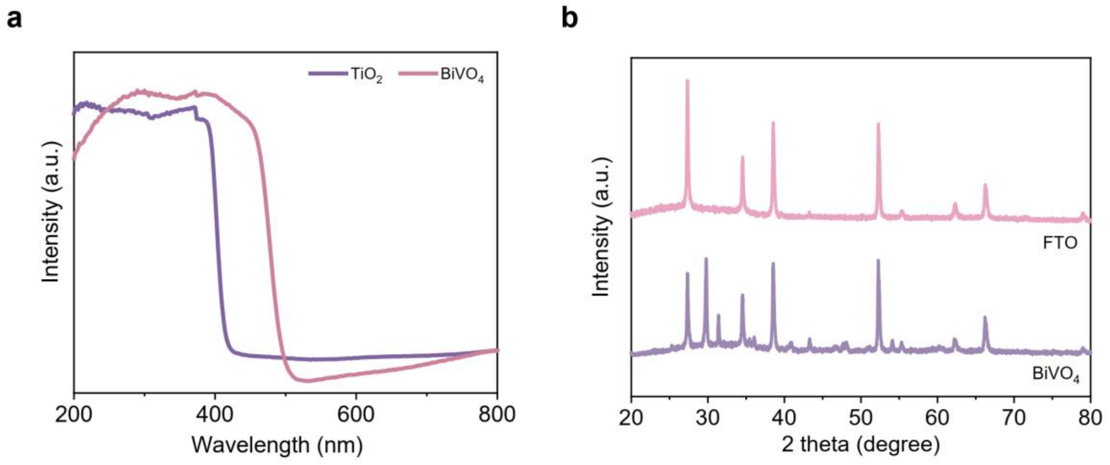

Supplementary Figure 6. Structural characterization of $BiVO_4$ photoanode: (a) Ultraviolet-visible absorption spectrum of TiO_2 and $BiVO_4$ film and (b) XRD spectrum of $BiVO_4$ film grown on a FTO conductive glass substrate.

The optical absorption range of TiO_2 is located in the ultraviolet region within 400 nm, while the optical absorption range of $BiVO_4$ is mainly located in the ultraviolet and visible region within 500 nm).

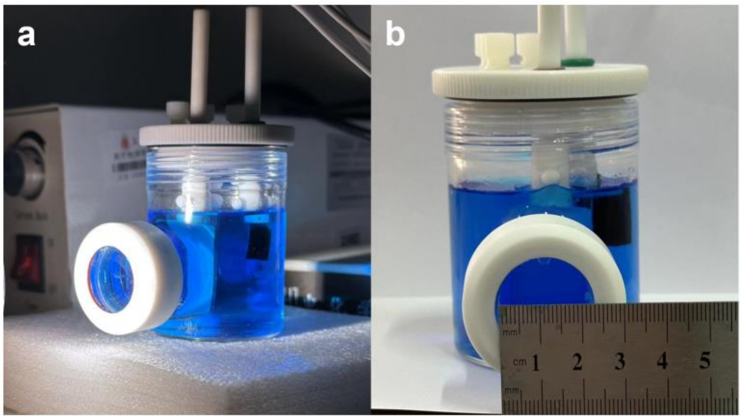

Supplementary Figure 7. Our IAPEC test unit (a)left view and (b) front view.

As shown in Supplementary Fig. 7, in the electrolytic cell used for our photochemical reactions. The window on the photoelectrode that receives light is approximately a circular area with a diameter of about 2.5 cm (The area approximately 4.91 cm^2).

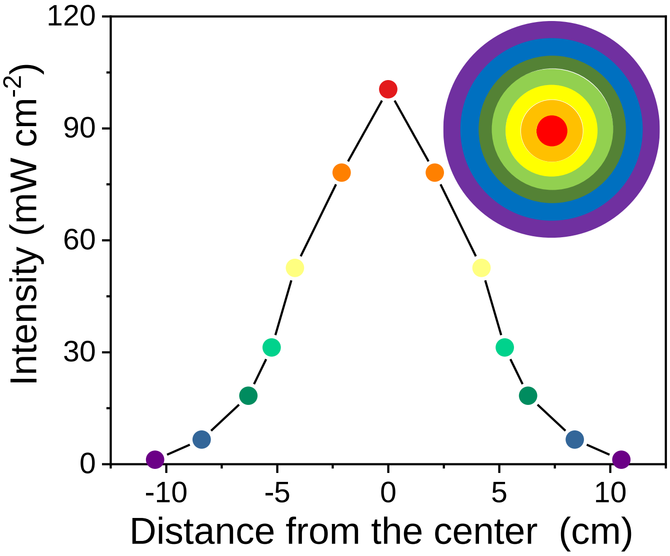

Supplementary Figure 8. Distribution of the irradiation intensity of the simulated sunlight.

As depicted in Supplementary Fig. 8, it can be observed that the irradiance gradually decreases from the center to the periphery. Hence, within approximately 1.5 cm from the center, the irradiance from the simulated light source can be greater than 90 mW cm^{-2}.

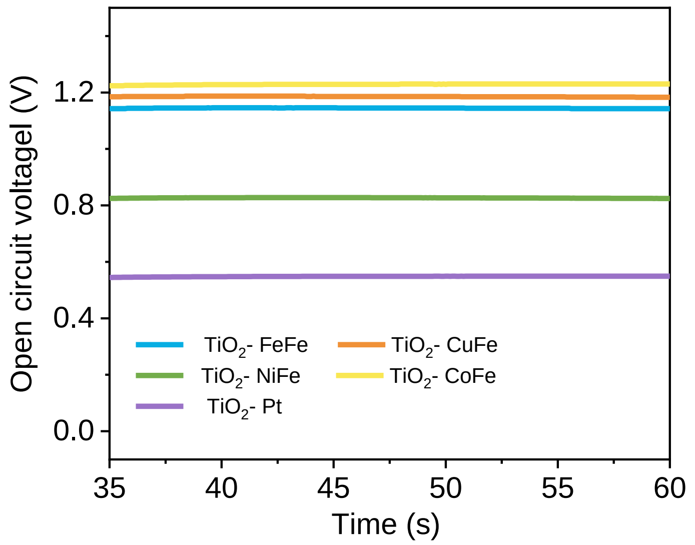

Supplementary Figure 9. Open circuit voltage (OPV) of different PEC systems.

As depicted in Supplementary Fig. 9, the IAPEC system produced significantly higher voltages of 1.15, 1.19, 0.8 and 1.12 V with different types of PBAs cathode, respectively, in comparison to the 0.55 V OPV obtained by the PEC (TiO_2-Pt) system.

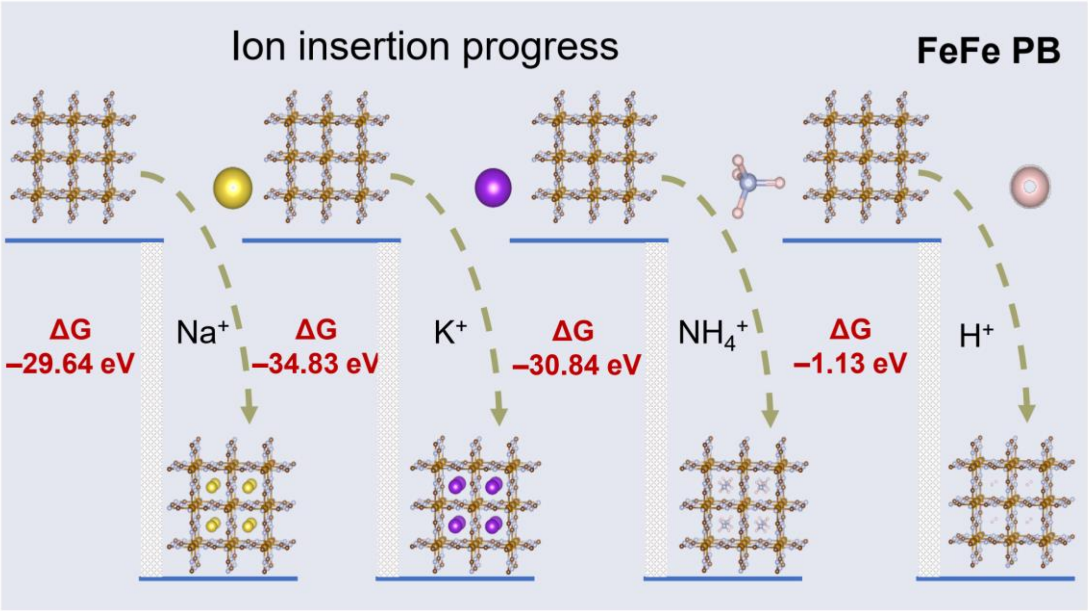

Supplementary Figure 10. The theoretical calculation of the Gibbs free energy change for the FeFe PB structure during the insertion process of Na^+, K^+ $NH_4^+$and H^+ ions.

As detailed in Supplementary Fig. 10, the free energy required for H^+ ions insertion by FeFe PB is –1.13 eV. Notably, this value is significantly higher than the energy barrier for the insertion of the other three cations, yet it remains lower than the energy barrier of 0.47 eV for the HER reaction. These results further support our work findings and enhance the relevance of the comparison.

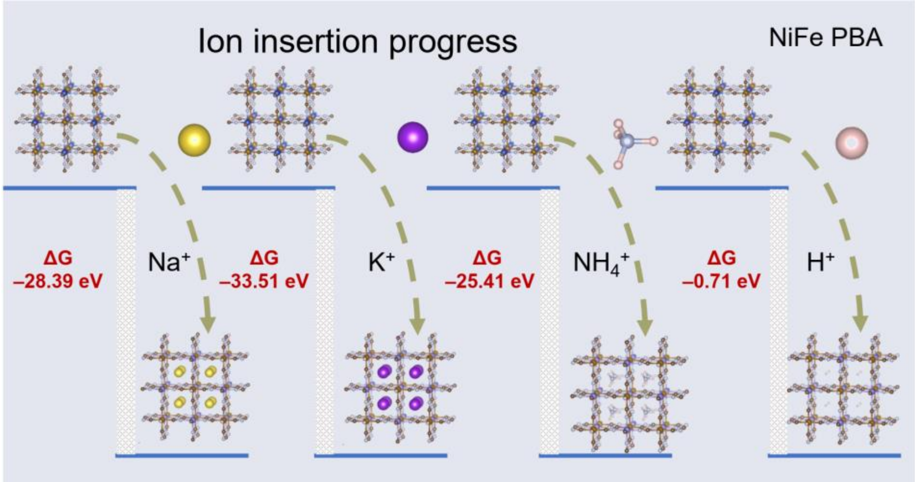

Supplementary Figure 11. The theoretical calculation of the Gibbs free energy change for the NiFe PBA structure during the insertion process of Na^+, K^+ $NH_4^+$and H^+ ions.

As detailed in Supplementary Fig. 11, the free energy required for H^+ ion insertion by NiFe PBA is –0.71 eV. Notably, this value is significantly higher than the energy barrier for the insertion of the other three cations, yet it remains lower than the energy barrier of 0.47 eV for the HER reaction. These results further support our work findings and enhance the relevance of the comparison.

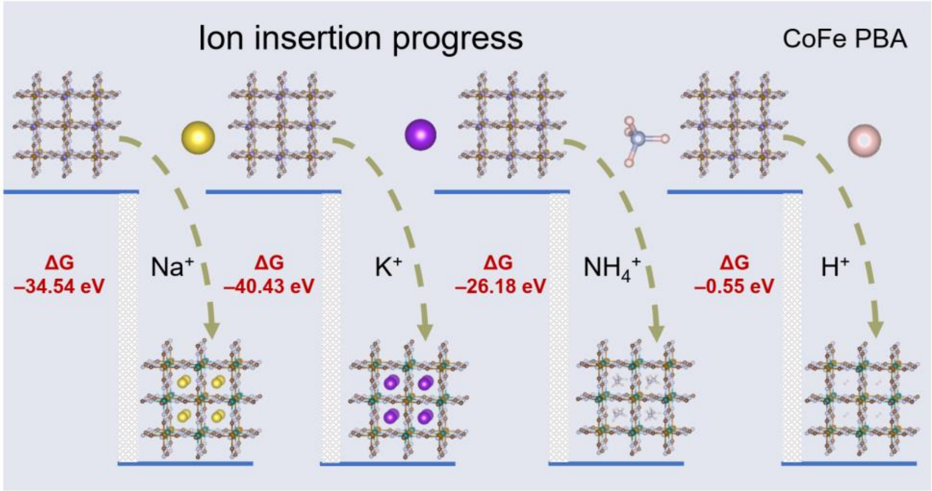

Supplementary Figure 12. The theoretical calculation of the Gibbs free energy change for the CoFe PBA structure during the insertion process of Na^+, K^+ $NH_4^+$and H^+ ions.

As detailed in Supplementary Fig. 12, the free energy required for H^+ ion insertion by CoFe PBA is –0.55 eV. Notably, this value is significantly higher than the energy barrier for the insertion of the other three cations, yet it remains lower than the energy barrier of 0.47 eV for the HER reaction. These results further support our work findings and enhance the relevance of the comparison.

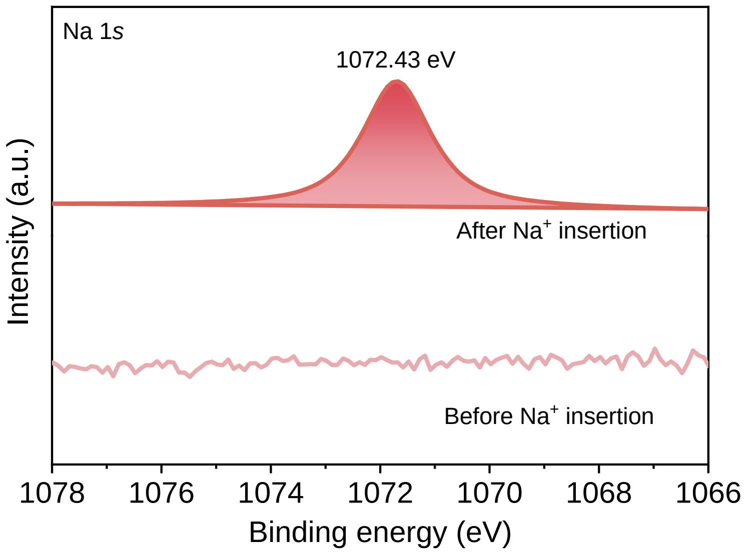

Supplementary Figure 13. The Na *1s* XPS spectra of CuFe PBA electrode before and after ion insertion in the IAPEC system.

X-ray photoelectron spectroscopy (XPS) analysis revealed the evolution of Na *1s* peak appeared at 1072.4 eV.

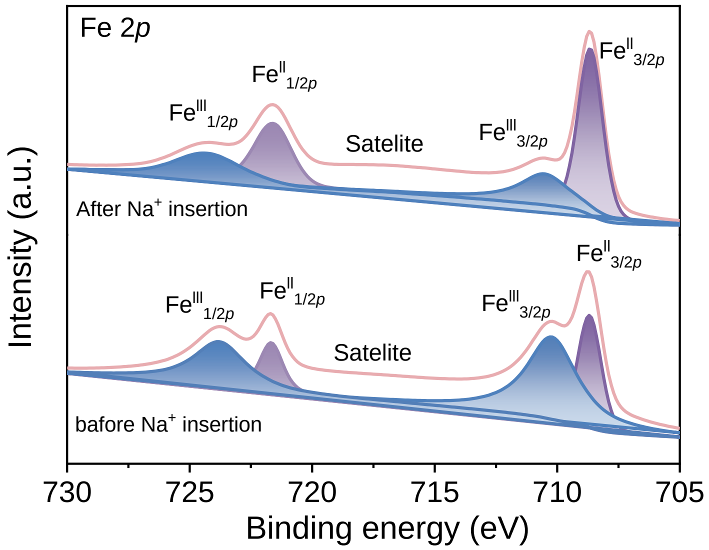

Supplementary Figure 14. High-resolution XPS spectra of Fe 2*p* in CuFe PBA before and after interaction with Na^+ ions.

The area of Fe $^{(III)}$ 2*p* region gradually decreases while the area of Fe $^{(II)}$ 2*p* region increases, indicating that most Fe $^{(III)}$ are reduced to Fe $^{(II)}$ after the Na^+ insertion processes of CuFe PBA.

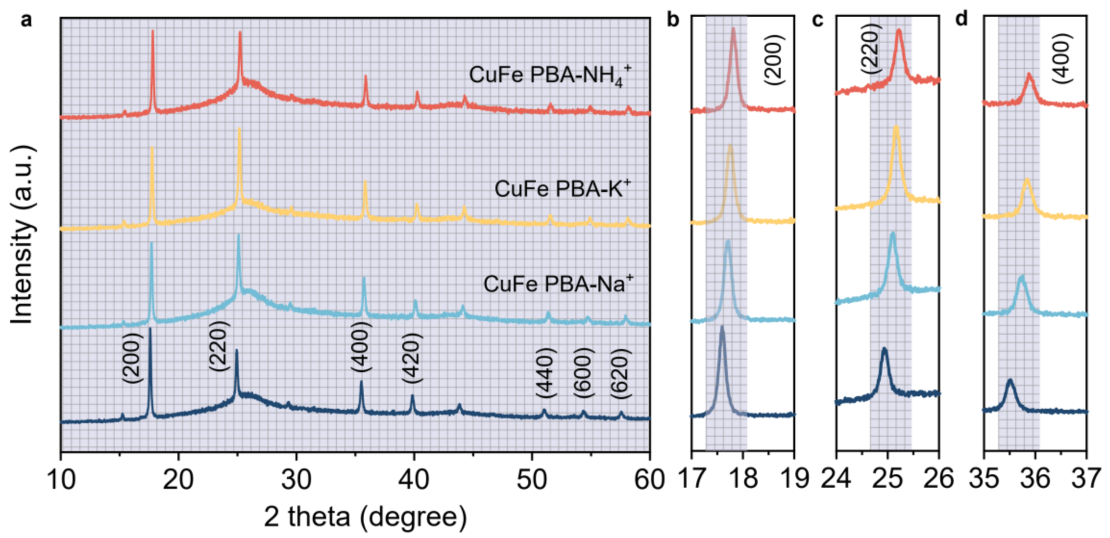

Supplementary Figure 15. (a) XRD patterns of CuFe PBA electrode before and after ion insertion in the IAPEC system (b) Enlarged view of the (200) crystal plane. (c) Enlarged view of the (220) crystal plane. (d) Enlarged view of the (400) crystal plane.

The crystal structure of CuFe PBA always maintains a face-centered cubic structure with the embedding of sodium ions, and the position of the diffraction peak shifts to a higher angle of the diffraction peak, indicating the slightly shrink of framework.

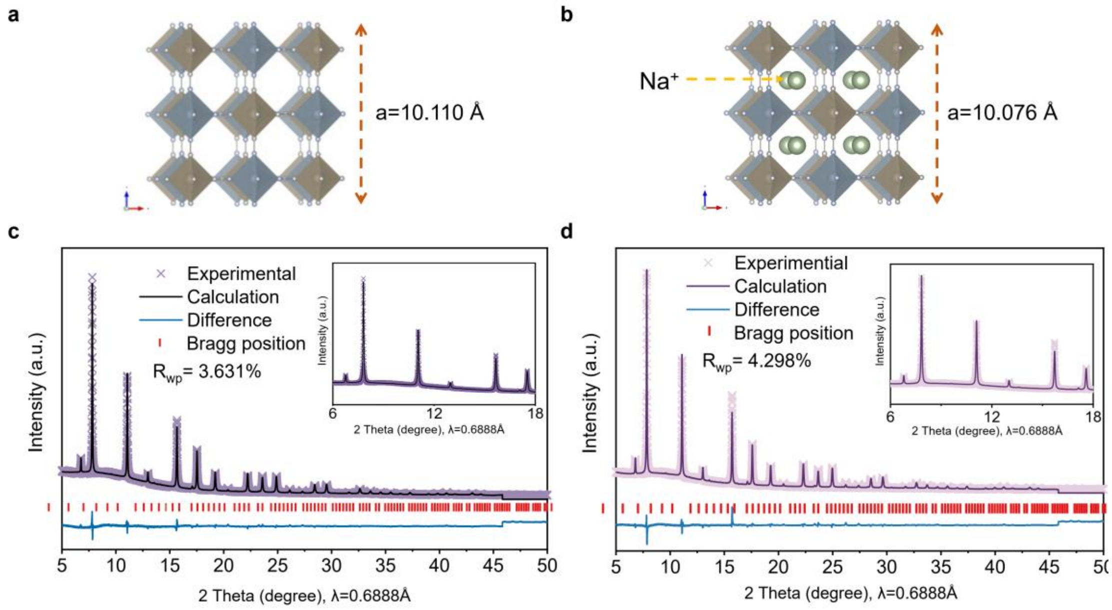

Supplementary Figure 16. (**a**) Schematic structure CuFe PBA sample before and (**b**) after Na^+ ions insertion. (**c**) Rietveld refinement synchrotron PXRD pattern of CuFe PBA sample before and (**d**) after Na^+ ions insertion.

Supplementary Fig. 16 depicts the refined crystal structure before and after the insertion of Na^+ ions. CuFe PBA retains its Fm-3m structure after insertion of Na^+ ions, leading to a change in the lattice parameter from 10.110 to 10.076 Å. This can be attributed to superior stability of CuFe PBA, which has an ultra-low strain open frame structure.

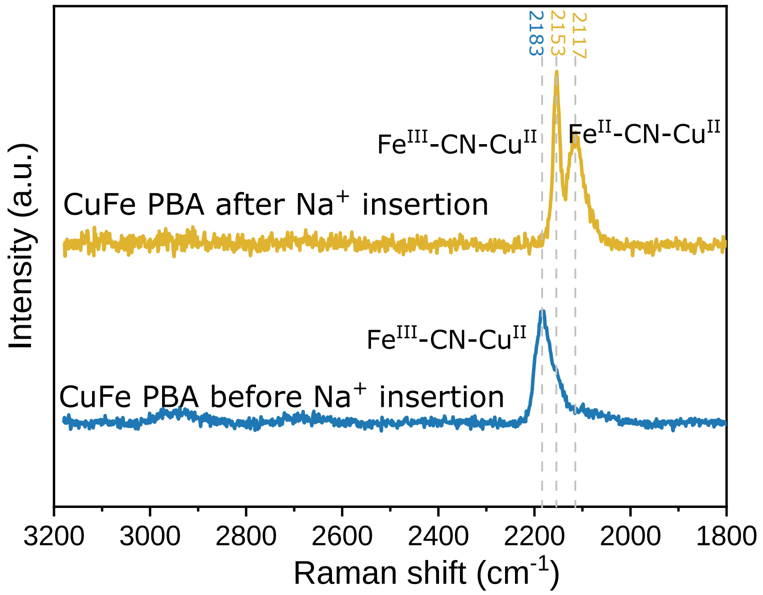

Supplementary Figure 17. Raman spectra of CuFe PBA sample before and after Na^{+} ions insertion.

Raman spectra characterization can be seen that after Na^{+} ions insertion, the characteristic peak of the cyanide shifted in lower wavenumber peaks. Due to the frequency of the cyanide stretching vibration mode is sensitive to the surrounding chemical environment, so the cyanide coordinated with Fe^{II} shows a relatively lower wavenumber peaks than the cyanide coordinated with Fe^{III}.

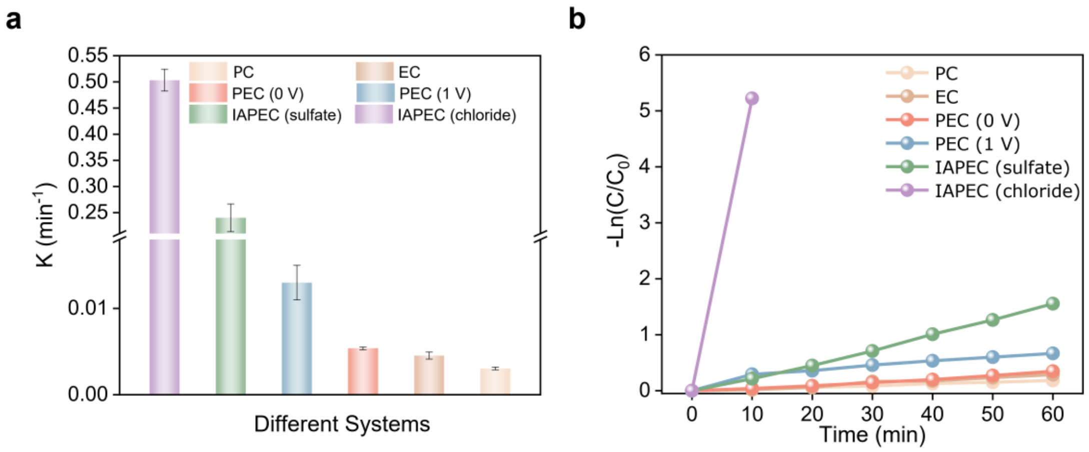

Supplementary Figure 18. (a) The degradation pseudo-first-order rate constant of IAPEC for MB by different systems. (b) Pseudo-first-order kinetic fitting models of MB degradation in different systems.

The corresponding pseudo-first-order rate constant of IAPEC in chloride medium is as high as 0.5 min^{-1} within 60 min, while that of IAPEC in Sulfate medium PEC(0 V), PEC(1 V), EC and PC are 0.24,0.12, 0.058,0.05 and 0.03 min^{-1}, respectively. Error bars representing the standard deviation of three replicate measurements.

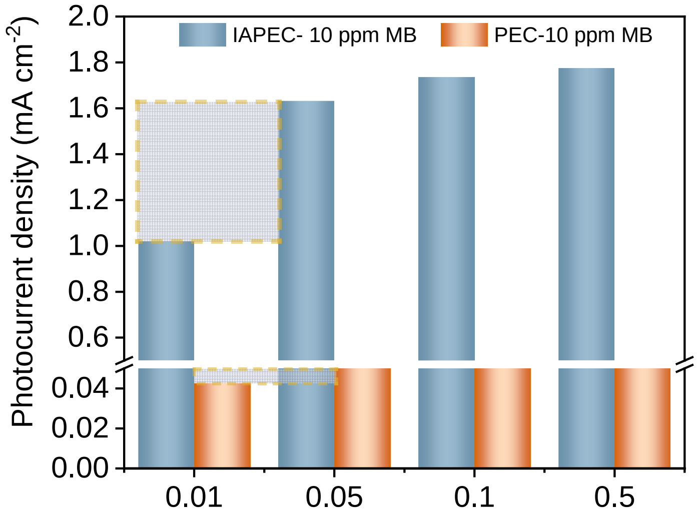

Supplementary Figure 19. Transient photocurrent densities of PEC and IAPEC systems under intermittent simulated sunlight irradiation in different concentrations (0.01 M to 0.5 M) of NaCl solution (10 ppm MB).

When the concentration of sodium chloride increased from 0.01 M to 0.5 M, the photocurrent density of the conventional PEC system increased from 0.04 to 0.05 mA cm^{-2}, while that of the IAPEC system increased from 1 to approximately 1.6 mA cm^{-2}.

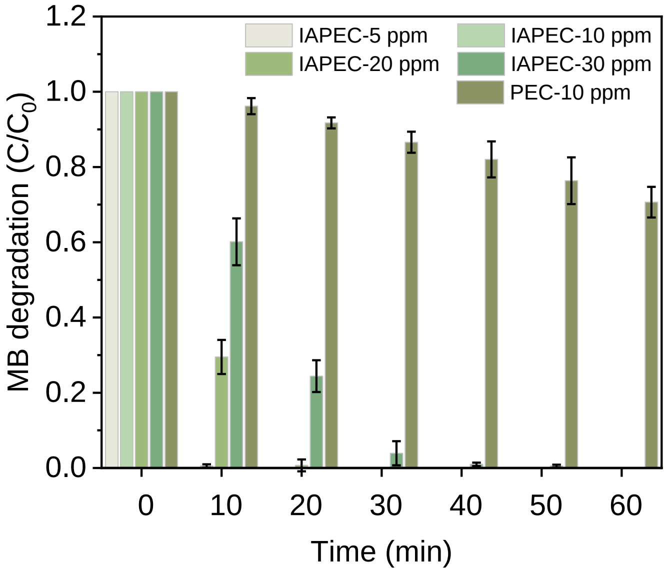

Supplementary Figure 20. Degradation of MB in different systems and concentrations (The MB concentration increased from 5 ppm to 30 ppm). Error bars representing the standard deviation of three replicate measurements.

Notably, as the MB concentration increased from 5 ppm to 30 ppm, the IAPEC system achieved remarkable performance by effectively removing over 99% of MB within 10 min

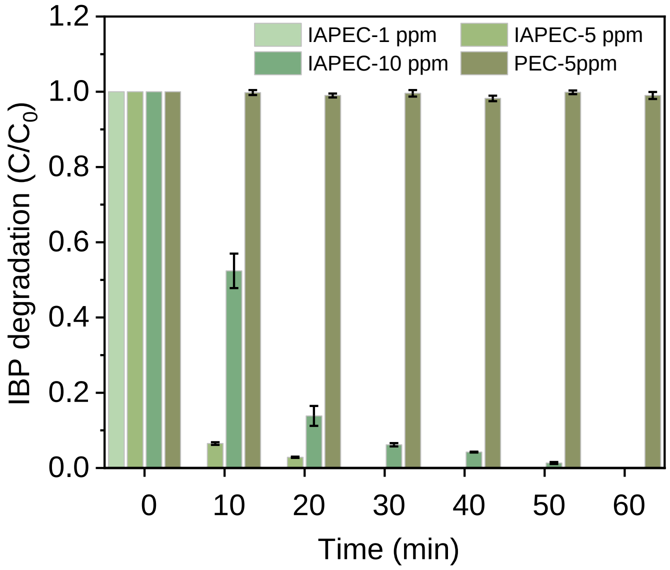

Supplementary Figure 21. Degradation of IBP in different systems and pollution concentrations (The IBP concentration increased from 5 ppm to 30 ppm). Error bars representing the standard deviation of three replicate measurements.

The IAPEC system achieved 99% degradation within 20 min as the IBP concentration increased from 1 ppm to 5 ppm. Even at a higher concentration of 10 ppm, the system still achieved over 90% degradation within 50 min. In contrast, the conventional PEC system exhibited only 1.8 % degradation rate for 10 ppm IBP after 60 min.

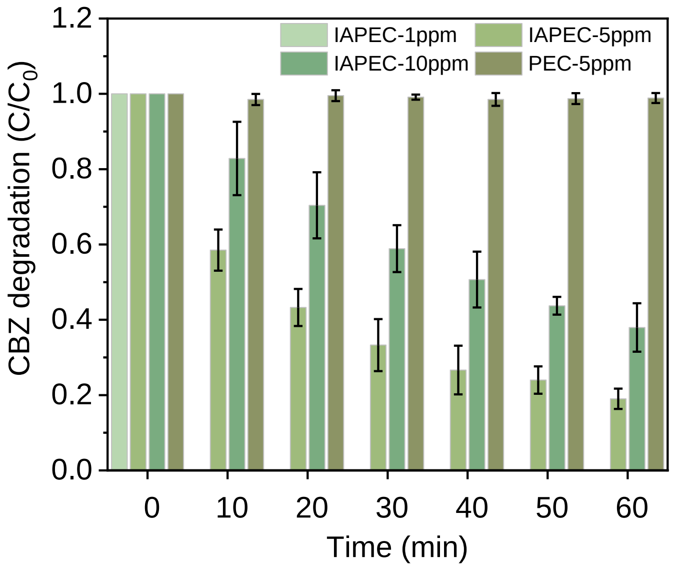

Supplementary Figure 22. Degradation of CBZ in different systems and concentrations (The CBZ concentration increased from 1 ppm to 10 ppm). Error bar representing the standard deviation of three replicate measurements.

Similarly, the IAPEC system achieved complete degradation of 1 ppm CBZ within 10 min, with degradation rates of 5 and 10 ppm CBZ reaching 81% and 63% within 1 h, respectively. In contrast, the PEC system displayed negligible degradation rates for CBZ.

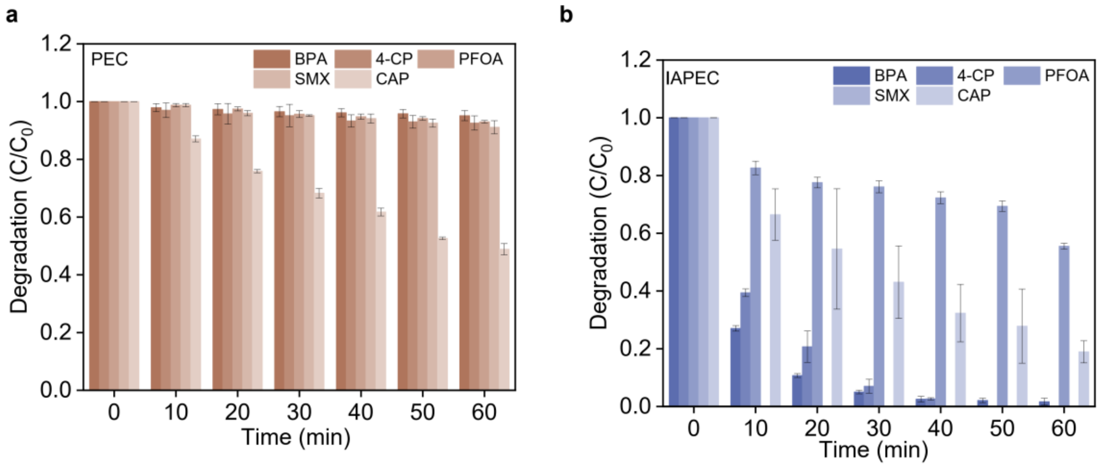

Supplementary Figure 23. The degradations performances of PEC (a) and IAPEC (b) systems for Bisphenol A (BPA), 4-Chlorophenol (4-CP), Perfluorooctanoic Acid (PFOA), Sulfamethoxazole (SMX) and Cellulose Acetate Propionate (CAP). (Experimental conditions: 60 mL 0.1 M NaCl solution, initial pH = 6.0 ± 0.1, simulated solar light illumination 100 mW cm^{-2}, pollutant concentration: 10 ppm). Error bars representing the standard deviation of three replicate measurements.

After a 60 min treatment in the PEC system, the degradation rate of other pollutants was basically ignored except for 50% degradation of CAP. In contrast, in the IAPEC system, except for PFOA, all other pollutants reached 80% degradation after 1 h treatment.

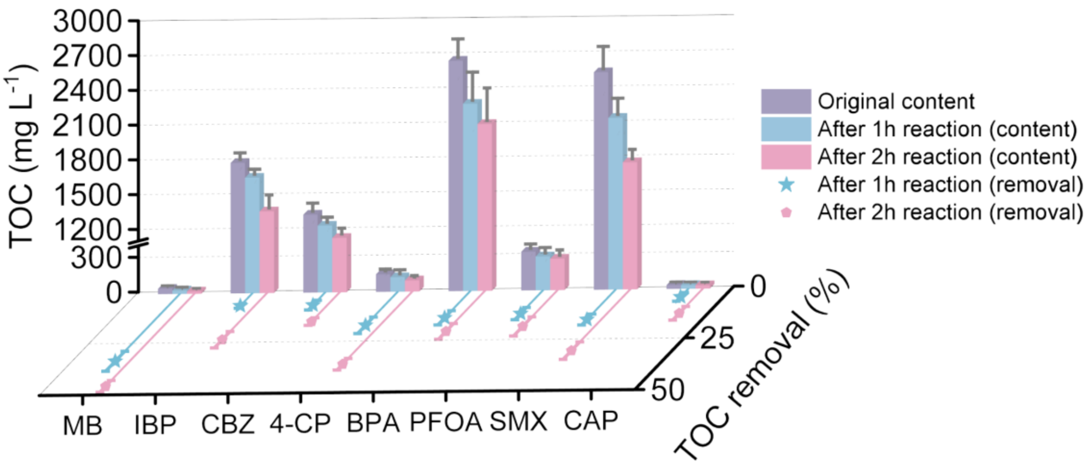

Supplementary Figure 24. The removal rate of TOC of eight model pollutants by IAPEC system after 2 h reaction. (Experimental conditions: 60 mL 0.1 M NaCl solution, initial pH = 6.0 ± 0.1, simulated solar light illumination 100 mW cm^{-2}, pollutant concentration: 10 ppm). Error bars representing the standard deviation of three replicate measurements.

The findings revealed that the IAPEC system achieves an impressive mineralization efficiency, reaching up to ~50%. Particularly significant is the observation that pollutants with higher organic carbon content, such as PFOA, also achieved a commendable mineralization efficiency of about 40%.

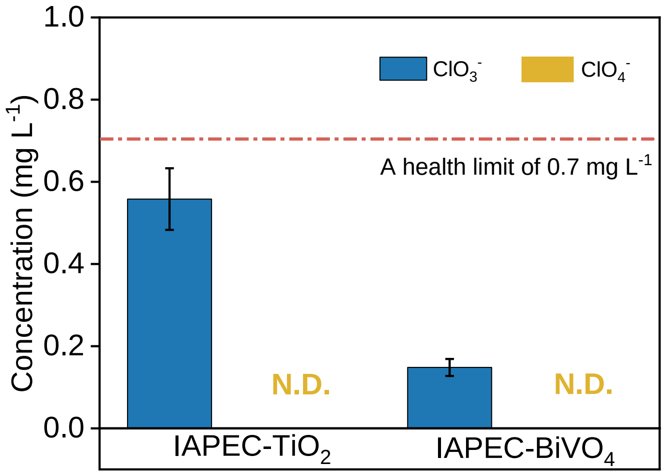

Supplementary Figure 25. Concentrations of toxic oxychlorides during the IAPEC treatment of saline sewage with different Photoanode. (N.D. is not detected, experimental conditions: 60 mL 50 mM NaCl simulated saline sewage, using TiO_2 or $BiVO_4$ as photoanode, conducted in triplicate). Error bars representing the standard deviation of three replicate measurements.

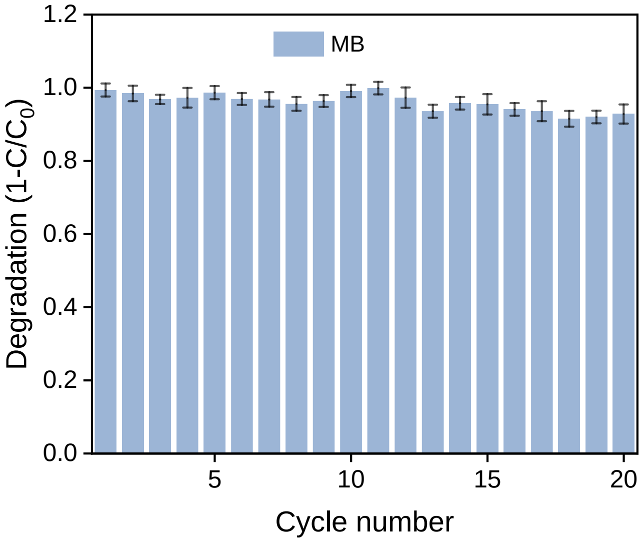

Supplementary Figure 26. Reusability performance of MB after 20 cycles (experimental conditions: 60 mL 0.1 M NaCl solution, initial pH = 6.0 ± 0.1, simulated solar light illumination 100 mW cm^{-2},, pollutant concentration: 10 ppm, degradation time: 10 min). Error bars representing the standard deviation of three replicate measurements.

Reusability performance is also as a vital indicator to evaluate the stability of the PEC system. As depicted in **Supplementary Fig. 26**, the degradation rate of MB only slightly decreased after 20 cycles but still remained high at 92.8%.

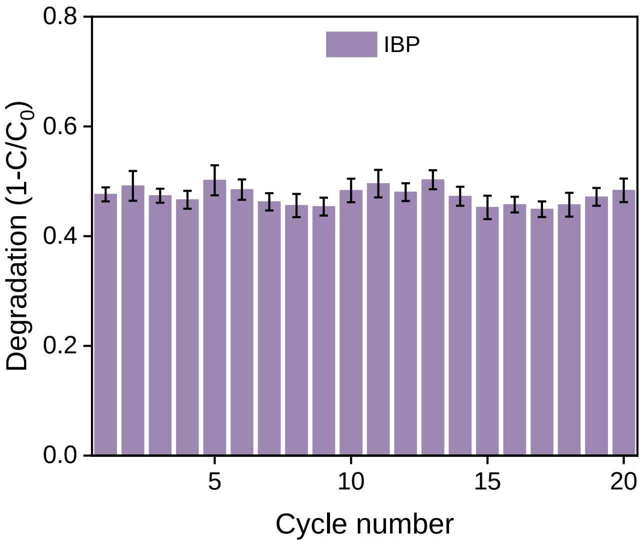

Supplementary Figure 27. Reusability performance of IBP after 20 cycles (experimental conditions: 60 mL 0.1 M NaCl solution, initial pH = 6.0 ± 0.1, simulated solar light illumination 100 mW cm^{-2}, pollutant concentration: 10 ppm, degradation time: 10 min). Error bars representing the standard deviation of three replicate measurements.

Reusability performance is also as a vital indicator to evaluate the stability of the PEC system. As depicted in **Supplementary Fig. 27**, the degradation rates of IBP were found to be 50% within 10 min. After 20 cycles, the degradation efficiencies of different pollutants decreased by less than 5% compared to the initial cycle, indicating that the IAPEC system has good cyclic stability for pollutant degradation.

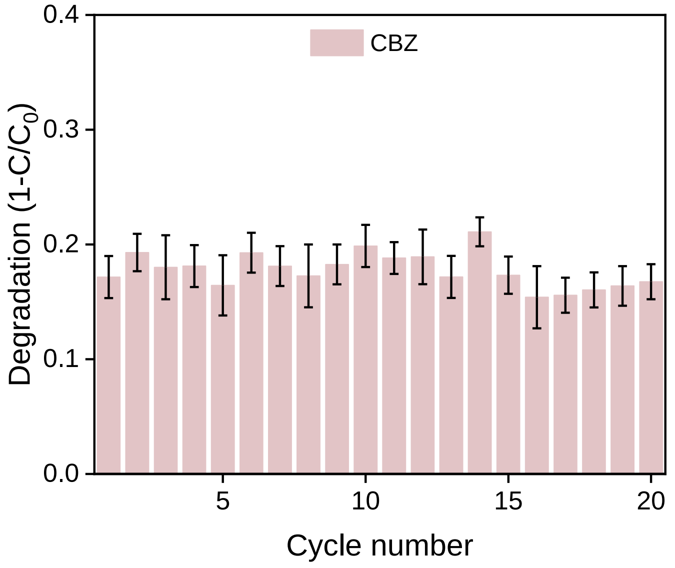

Supplementary Figure 28. Reusability performance of CBZ after 20 cycles (experimental conditions: 60 mL 0.1 M NaCl solution, initial pH = 6.0 ± 0.1, simulated solar light illumination 100 mW cm^{-2}, pollutant concentration: 10 ppm, degradation time: 10 min). Error bars representing the standard deviation of three replicate measurements.

Reusability performance is also as a vital indicator to evaluate the stability of the PEC system. As depicted in **Supplementary Fig. 28**, the degradation rates of CBZ were found to be 20% within 10 min. After 20 cycles, the degradation efficiencies of different pollutants decreased by less than 5% compared to the initial cycle, indicating that the IAPEC system has good cyclic stability for pollutant degradation.

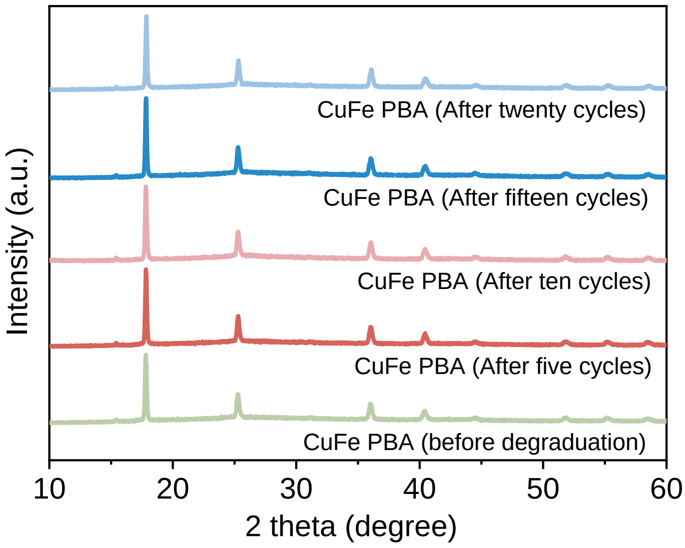

Supplementary Figure 29. X-ray diffraction patterns of CuFe PBA electrode after 20 cycles. (The test is performed five times per cycle).

The XRD patterns of the PBA electrode used for cycle experiment were found to be similar to those of the initial sample.

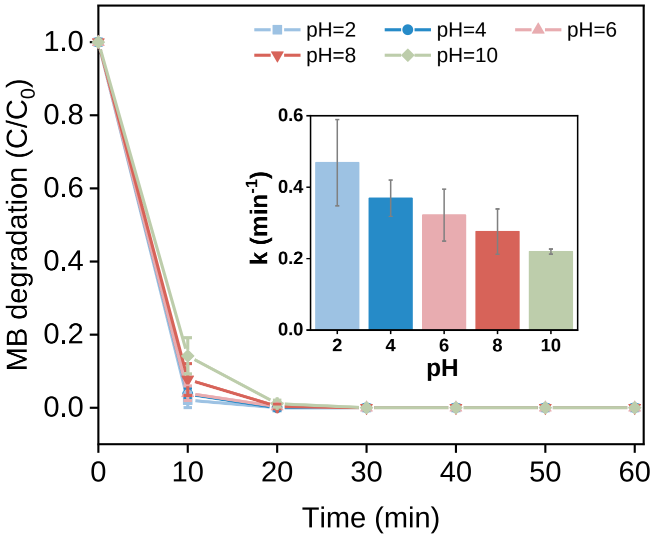

Supplementary Figure 30. The influence of pH variations on the MB degradation performance of the IAPEC system. (experimental conditions: 60 mL 0.1 M NaCl solution, initial pH = 6.0 ± 0.1, simulated solar light illumination 100 mW cm^{-2}). Error bars representing the standard deviation of three replicate measurements.

The pseudo-first-order rate constant of MB degradation decreases from 0.48 to 0.21 min^{-1} as pH value increases from 2 to 10.

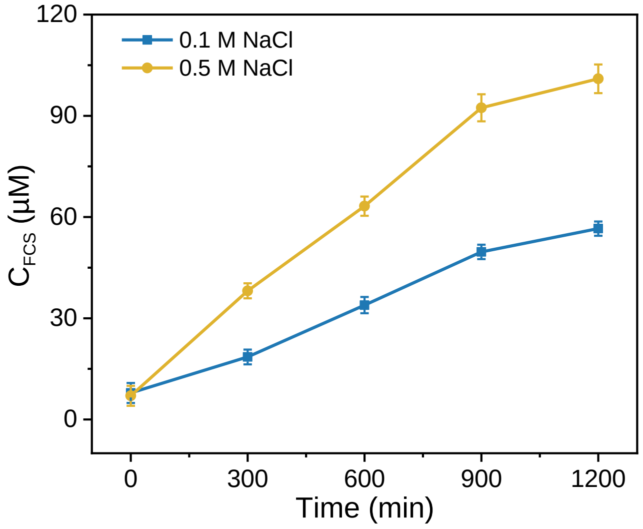

Supplementary Figure 31. The generation of free chlorine (FCS: Cl_2, HClO/ClO) during MB degradation in the IAPEC system. Error bars representing the standard deviation of three replicate measurements.

The decrease in pH is mainly due to the hole oxidation of H_2O and chloride ions resulting in the production of hydrogen ions and free chlorine species (FCS, Cl_2, HClO/ClO)

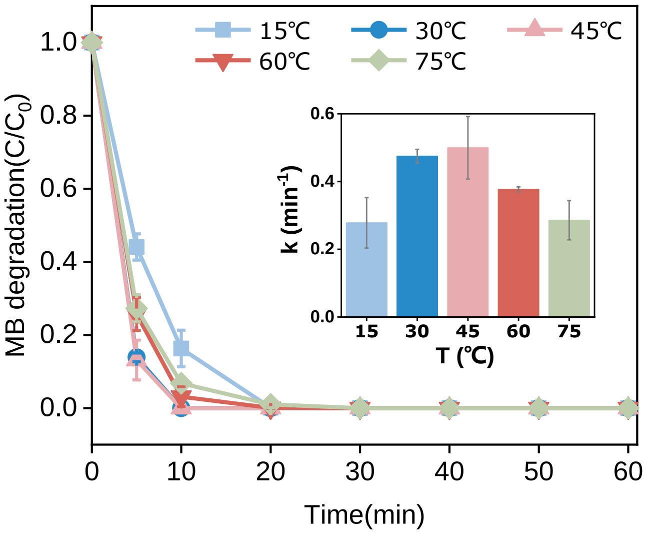

Supplementary Figure 32. The influence of temperature variations on the degradation performance of the IAPEC system. The insert is pseudo-first-order rate constant. (experimental conditions: 60 mL 0.1 M NaCl solution, initial pH = 6.0 ± 0.1, simulated solar light illumination 100 mW cm^{-2}). Error bars representing the standard deviation of three replicate measurements.

Apart from pH, as the temperature of the simulated wastewater increases from 15°C to 75°C, the degradation rate of MB exhibits a trend of initially increasing and then decreasing

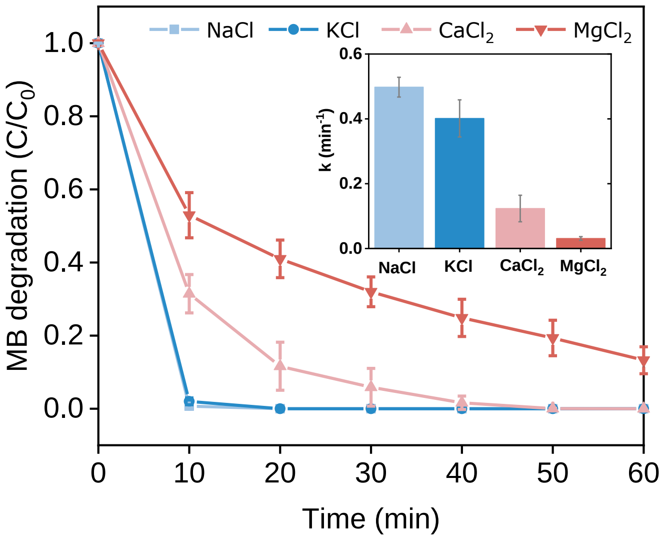

Supplementary Figure 33. The influence of NaCl, KCl, $CaCl_2$ and $MgCl_2$ on the degradation performance of the IAPEC system. The insert is pseudo-first-order rate constant. (experimental conditions: 60 mL 0.1 M NaCl solution, initial pH = 6.0 ± 0.1, simulated solar light illumination 100 mW cm^{-2}). Error bars representing the standard deviation of three replicate measurements.

The pseudo-first-order rate constants of MB degradation under the four electrolytes were NaCl (0.5 min^{-1}) > KCl (0.4 min^{-1}) > $CaCl_2$ (0.1 min^{-1}) > $MgCl_2$ (0.2 min^{-1}).

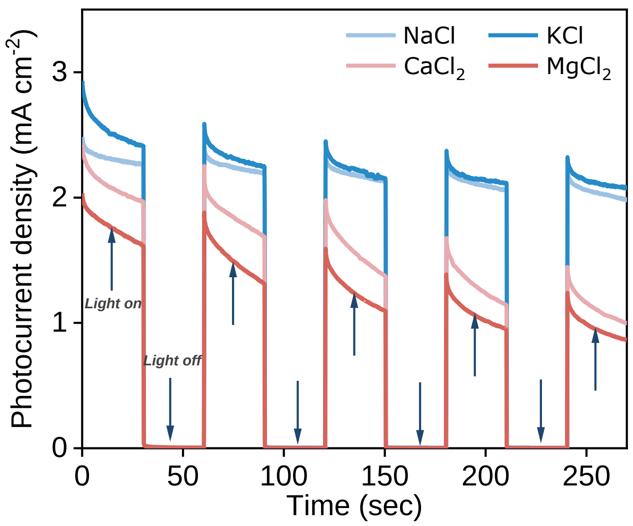

Supplementary Figure 34. The transient photocurrent density recorded for IAPEC system with and without sunlight irradiation in different electrolyte of 0.1 M NaCl, 0.1 M KCl, 0.05 M $CaCl_2$, 0.05 M $MgCl_2$. simulated solar light illumination 100 mW cm^{-2} xenon lamp source at 30 s intervals throughout a 270 s running.

The obtained results indicate that the photocurrent in the potassium ion solution is slightly higher than that in the sodium ion solution, which is consistent with the calculated Gibbs free energy results.

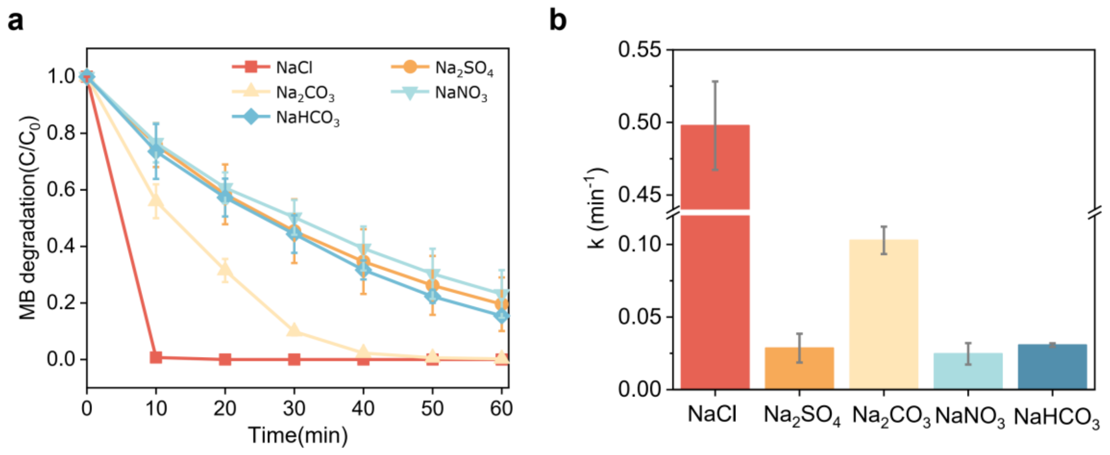

Supplementary Figure 35. (a) Degradation effect of different salts on MB and (b) Pseudo-first-order rate constant (experimental conditions: 60 mL simulated saline sewage, initial pH = 6.0 ± 0.1, simulated solar light illumination 100 mW cm^{-2}, [NaCl] = 0.1 M, [Na_2SO_4] = 0.1 M, [Na_2CO_3] = 0.1 M, [$NaNO_3$] = 0.1 M, [$NaHCO_3$] = 0.1 M, $[MB]_0$ = 10 ppm). Error bars representing the standard deviation of three replicate measurements.

As shown in **Supplementary Fig. 35**, after 60 min of degradation at the same concentration, the NaCl medium system exhibited the fastest degradation rate, with a pseudo-first-order rate constant of 0.5 min^{-1}, which was about 20 times higher than that of Na_2SO_4 (0.025 min^{-1}) and $NaNO_3$ (0.024 min^{-1}), respectively. This phenomenon could be attributed to the interaction between chloride ions and the generated holes, leading to the generation of highly reactive chlorine species.

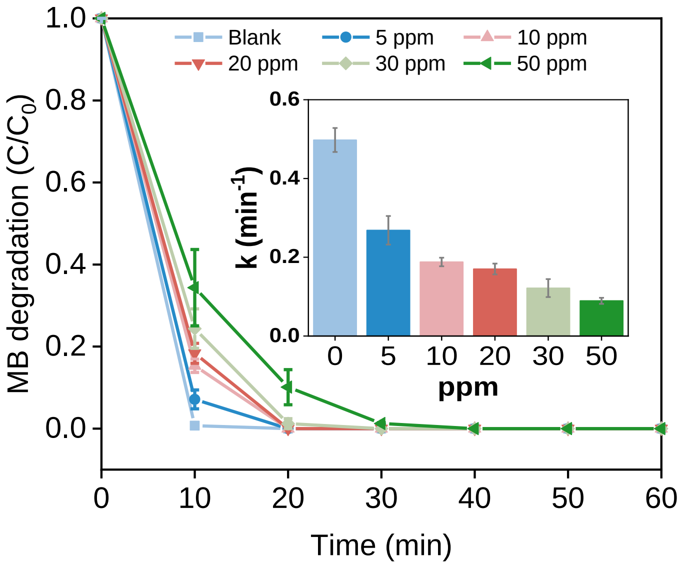

Supplementary Figure 36. The influence of the effects of humic acid (HA) on the degradation performance of the IAPEC system. The insert is pseudo-first-order rate constant. (Experimental conditions: 60 mL 0.1 M NaCl solution, initial pH = 6.0 ± 0.1, simulated solar light illumination 100 mW cm^{-2}). Error bars representing the standard deviation of three replicate measurements.

The results indicat that humic acid (HA) inhibited MB degradation in IAPEC system.

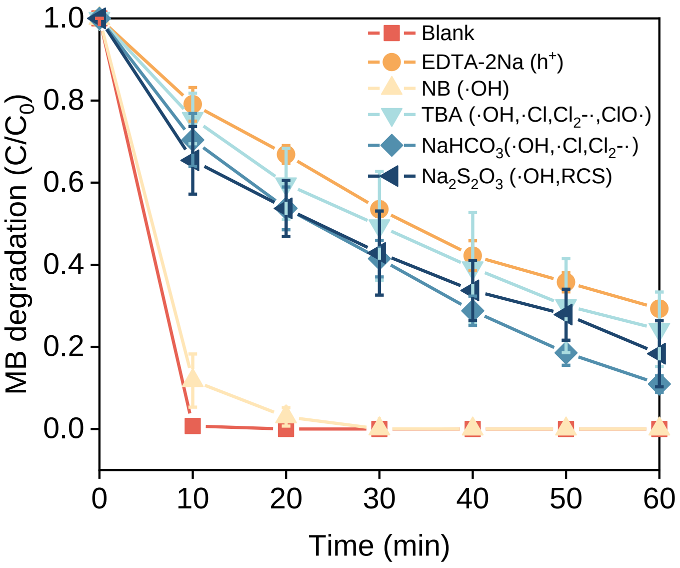

Supplementary Figure 37. The different free radical scavengers quenched. (The species and signal intensity of free radicals were determined by electron spin resonance spectroscopy (ESR). Quenching experiments were carried out with tert–butanol (TBA, •OH, Cl•, Cl_2-•, ClO•), $NaHCO_3$ (•OH, Cl•, Cl_2-•), nitrobenzene (NB, •OH), $Na_2S_2O_3$(•OH, Cl•, Cl_2-•, ClO•, HClO) and EDTA-2Na (h^+)). Error bars representing the standard deviation of three replicate measurements.

There was almost no change in the degradation rate after quenching •OH with NB (nitrobenzene), indicating that the main reactive species involved in degradation was not •OH. After adding TBA, the degradation rate of MB was significantly slow, indicating that the degradation process was mainly involved by chlorine free radicals.

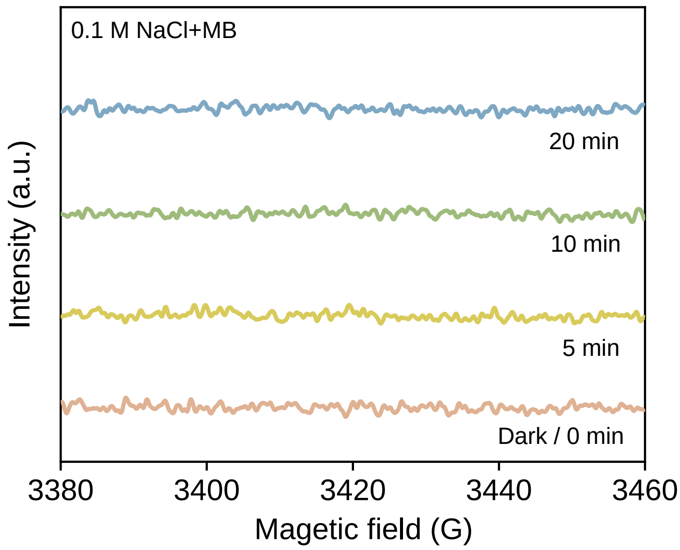

Supplementary Figure 38. ESR spectra of •Cl, and •Cl_2 in the PEC system with DMPO as a spin trap. In PEC system, TiO_2 and platinum sheet electrodes were used as photoanode and counter electrode, respectively, and 0.1 M NaCl was used as electrolyte. (The instrument is set as the modulation frequency: 100.00 KHz; Modulation amplitude: 2.00G; Scanning width: 100.00G. Scanning time: 80.720 s； the microwave power is 10.03 mW and the frequency is 9.857 GHz).

No free radicals were observed in dark conditions or in the conventional PEC system.

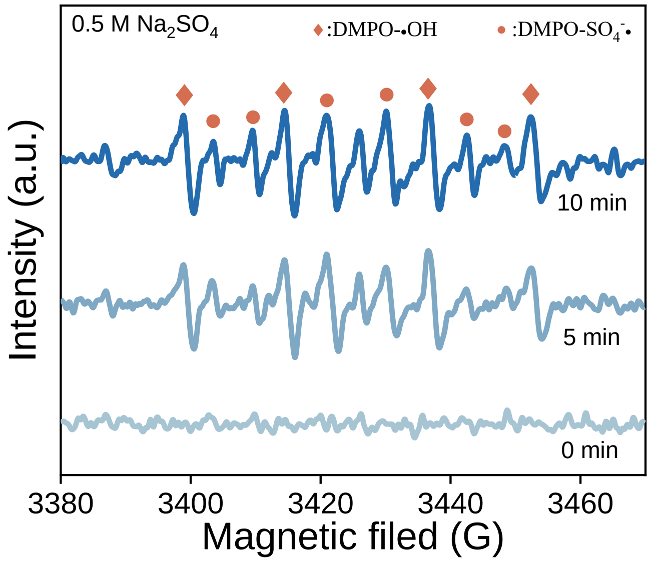

Supplementary Figure 39. ESR spectra of DMPO-·OH and DMPO-·SO_4^- adducts under sulfate media in IAPEC system.

It was indicating a consistent generation rate of ·OH and ·SO_4^-in our system.

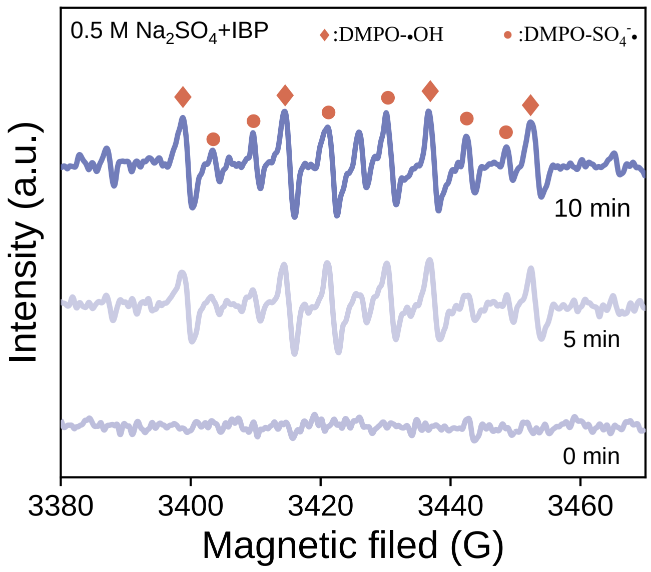

Supplementary Figure 40. ESR spectra of DMPO-·OH and DMPO-·SO_4^- adducts in sulfate media with 10 ppm IBP in IAPEC system.

Notably, even with the introduction of pollutants, the detected free radical signals remained relatively stable, indicating a consistent generation rate of ·OH and ·SO_4^-in our system.

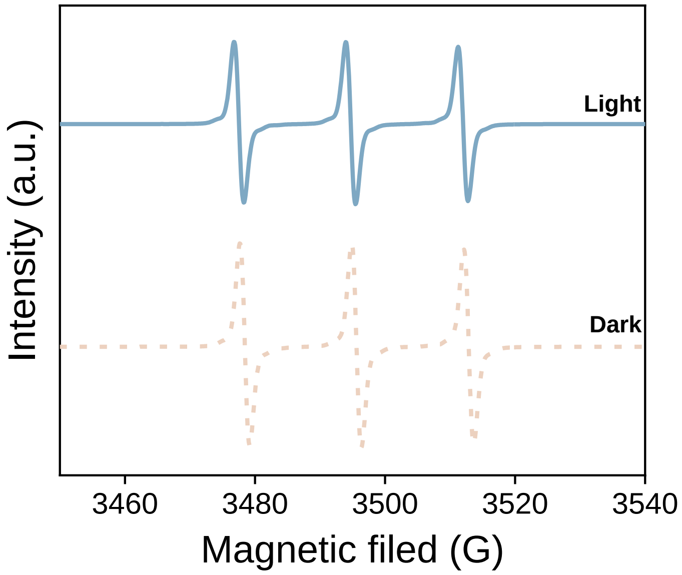

Supplementary Figure 41. ESR spectra of TEMP-h^+ adduct in sulfate media in our IAPEC system.

As shown in Supplementary Fig. 41, the typical 1:1:1 triplet signal is in good agreement with the TEMP signal. After illumination, a significant decrease in the triplet signal was observed, confirming the high generation of h^+ in our IAPEC system.

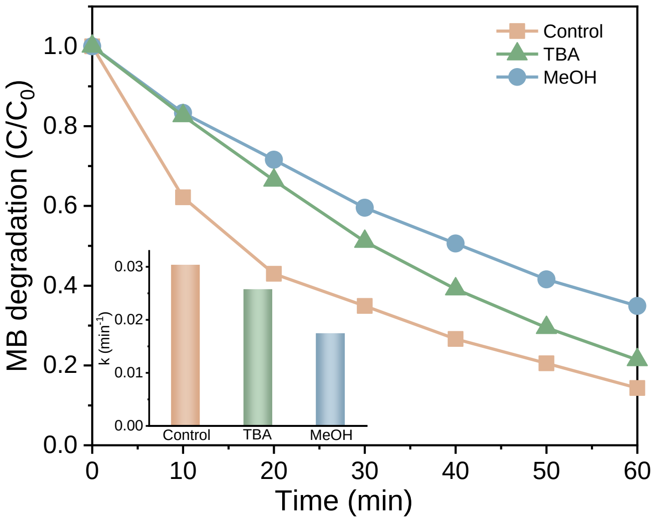

Supplementary Figure 42. The effect of quenchers on MB (10 ppm) degradation and the pseudo-first-order rate constants for quenching experiments in sulfate media in IAPEC system (inset picture). [TBA] and [MeOH] = 200 mM, [Na_2SO_4] = 0.5 M.

As shown in Supplementary Fig. 42, the addition of quenchers modestly inhibited the removal of MB (Methylene Blue), showcasing a 7%~20.6% reduction.

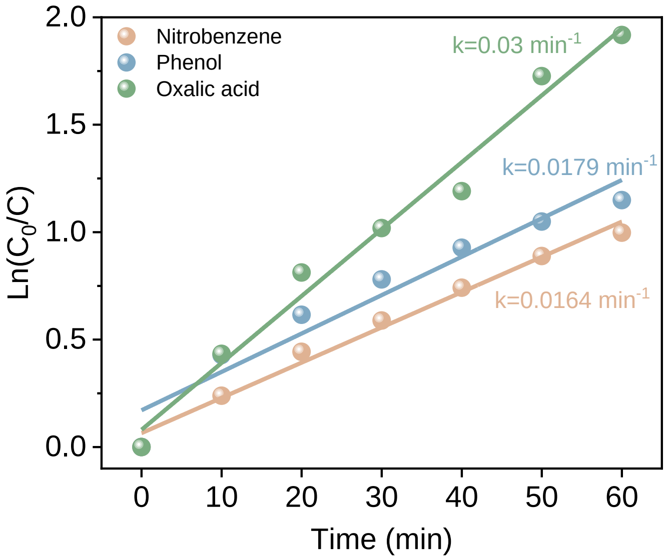

Supplementary Figure 43. Corresponding first-order kinetic curves of probe experiments for IAPEC system in sulfate media. [OA] = 100 mg L^{-1}, [Phenol] and [NB] as probe = 10 mg L^{-1}. [Na_2SO_4] = 0.5 M

The results in **Supplementary Fig. 43** showed that the degradation rate of oxalic acid (0.03 min^{-1}) was about twice that of nitrobenzene (0.0164 min^{-1}) and phenol (0.0179 min^{-1}).

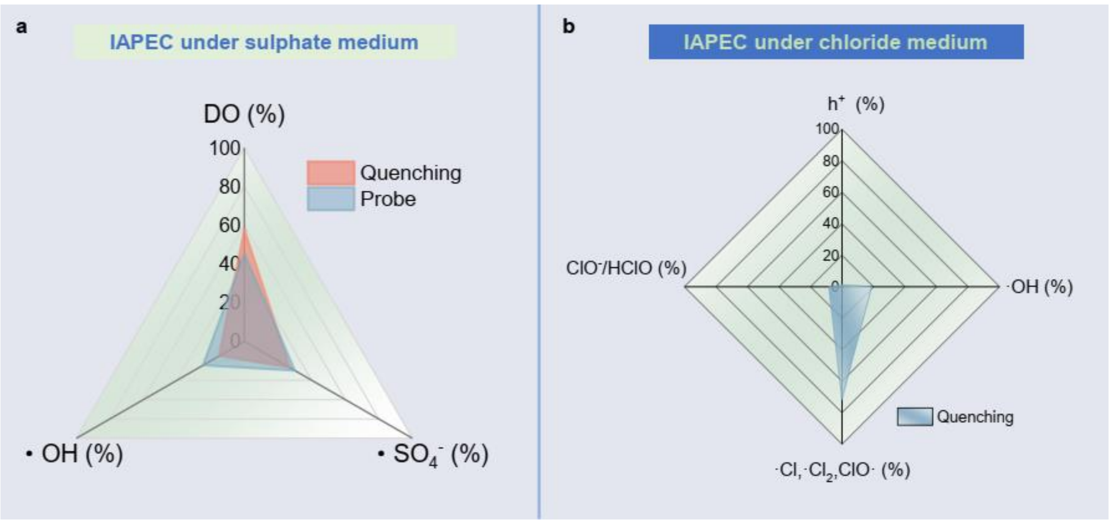

Supplementary Figure 44. Comparison of contributions of active species calculated based on quenching and probe experiments (a) IAPEC under sulphate medium. (b) IAPEC under chloride medium.

In summary, in the IAPEC system, the use of chloride salt as the electrolyte may produce more chlorine-containing groups and OH• co-degrade organic pollutants, while in the sulfate electrolyte, h^+ may mainly directly degrade organic pollutants.

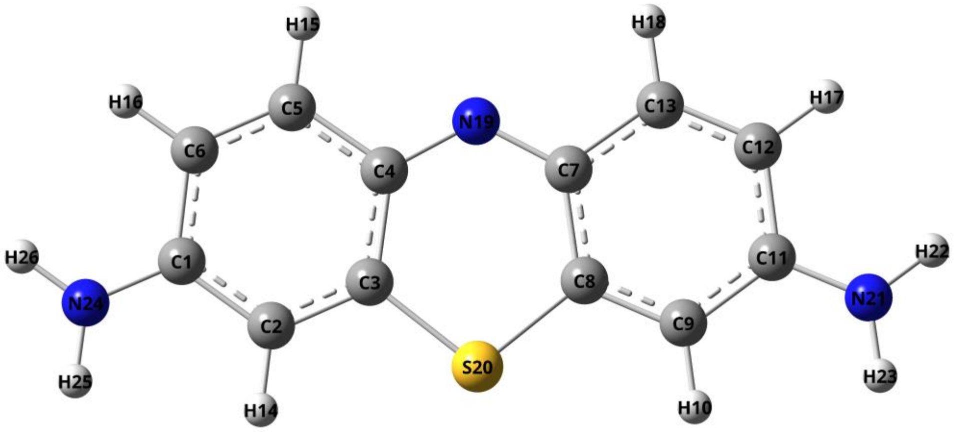

Supplementary Figure 45. Fukui function configuration analysis of $C_{12}H_{10}N_3S^+$ molecular configuration after optimization (with numbering).

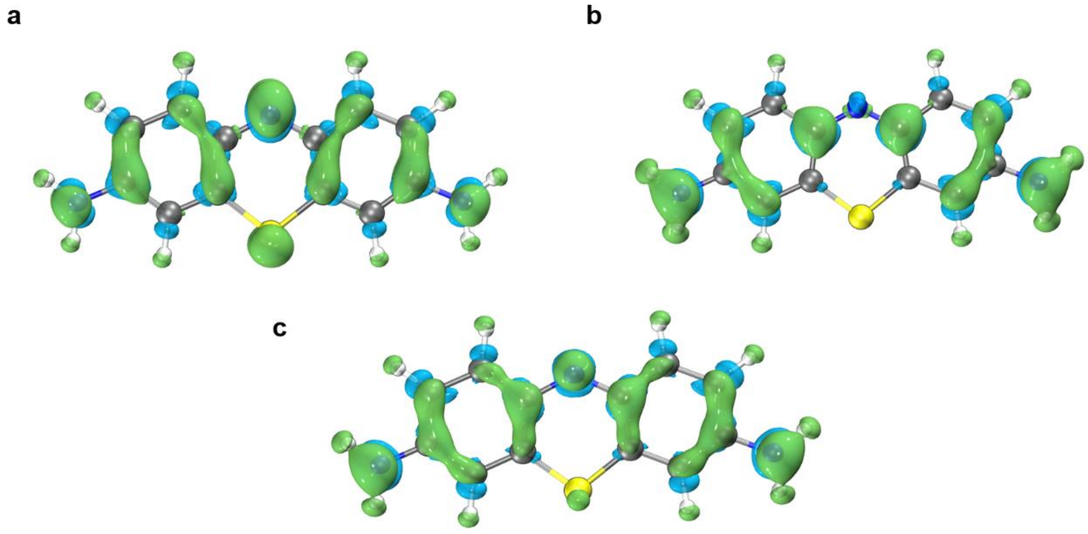

Supplementary Figure 46. Isosurface diagram of Fukui function of $C_{12}H_{10}N_3S^+$ molecule: (a) Electrophilic offense index f^+, (b) Nucleophilic aggression index f^-, (c) Free radical attack index f^0.

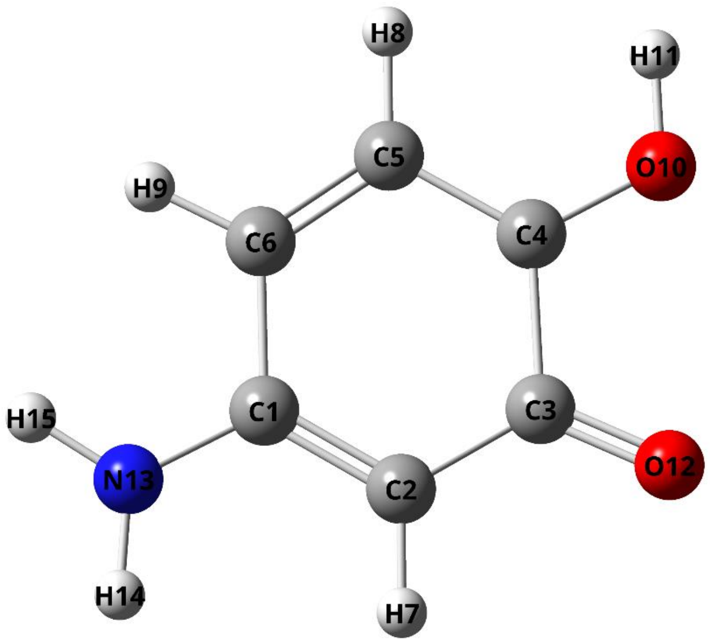

Supplementary Figure 47. Isosurface diagram of Fukui function of $C_6H_6NO_2^+$ molecular configuration after optimization (with numbering).

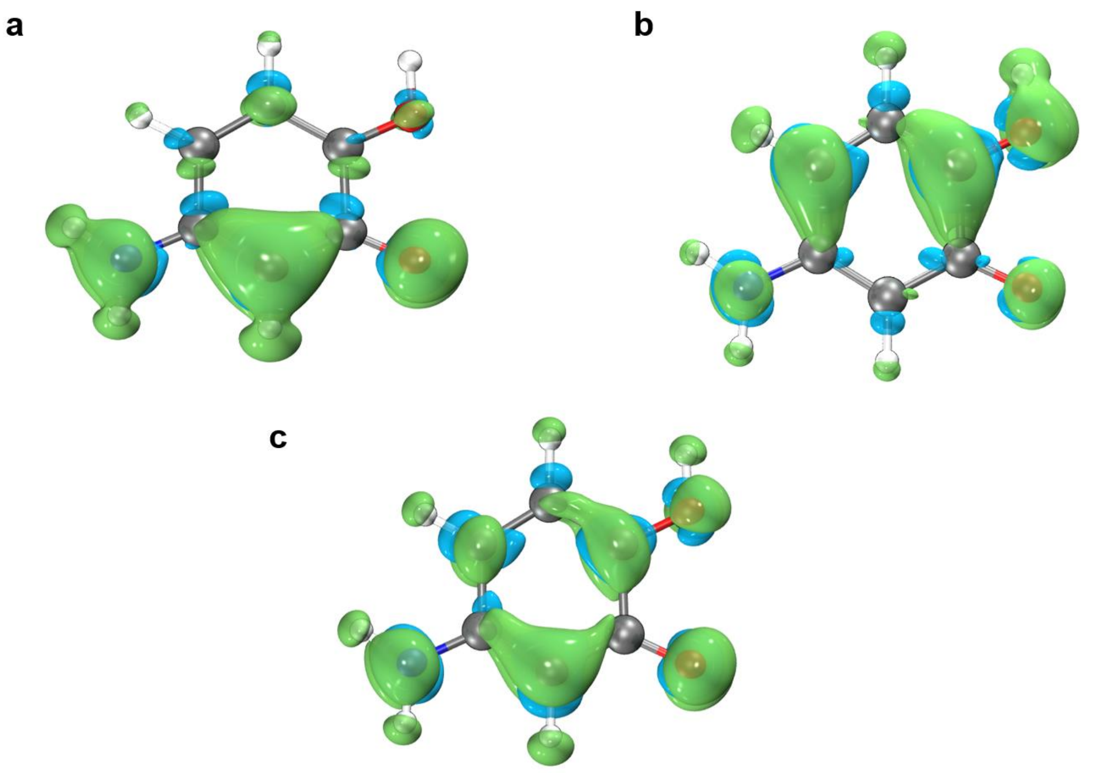

Supplementary Figure 48. Isosurface diagram of Fukui function of $C_6H_6NO_2^+$ molecule: (a) Electrophilic offense index f^-, (b) Nucleophilic aggression index f^+, (c) Free radical attack index f^0.

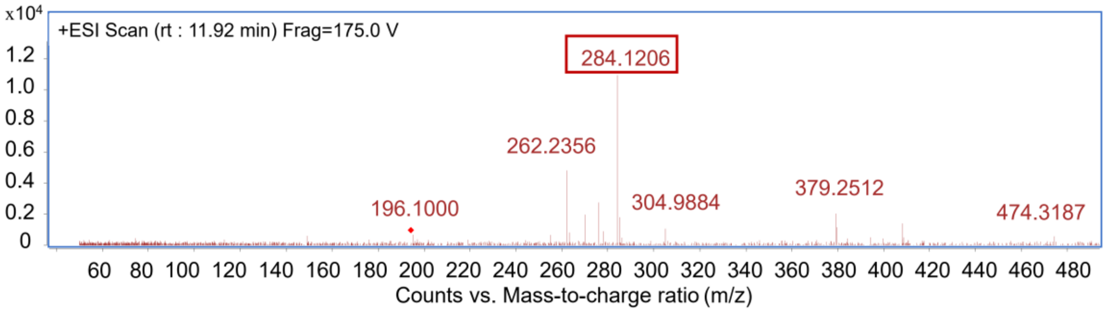
+ESI Scan (rt : 11.92 min) Frag=175.0 V
284.1206
262.2356
196.1000
304.9884
379.2512
474.3187
Counts vs. Mass-to-charge ratio (m/z)

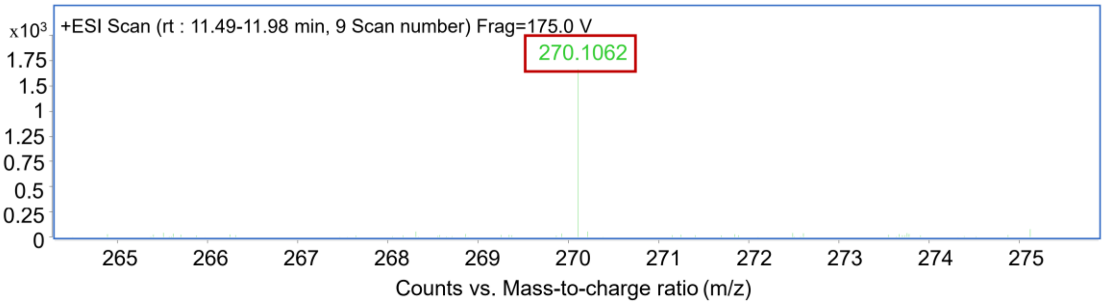
+ESI Scan (rt : 11.49-11.98 min, 9 Scan number) Frag=175.0 V
270.1062
Counts vs. Mass-to-charge ratio (m/z)

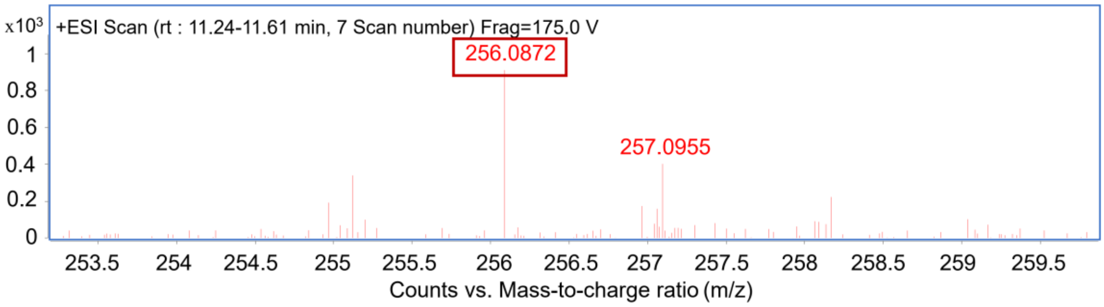
+ESI Scan (rt : 11.24-11.61 min, 7 Scan number) Frag=175.0 V
256.0872
257.0955
Counts vs. Mass-to-charge ratio (m/z)

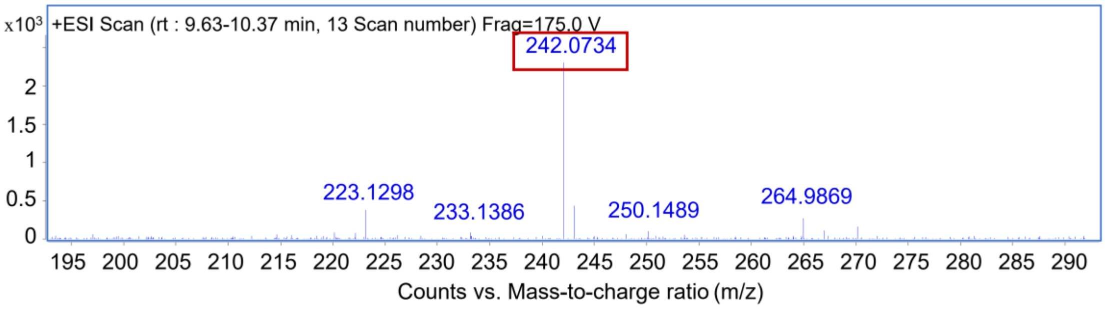
+ESI Scan (rt : 9.63-10.37 min, 13 Scan number) Frag=175.0 V
242.0734
223.1298
233.1386
250.1489
264.9869
Counts vs. Mass-to-charge ratio (m/z)

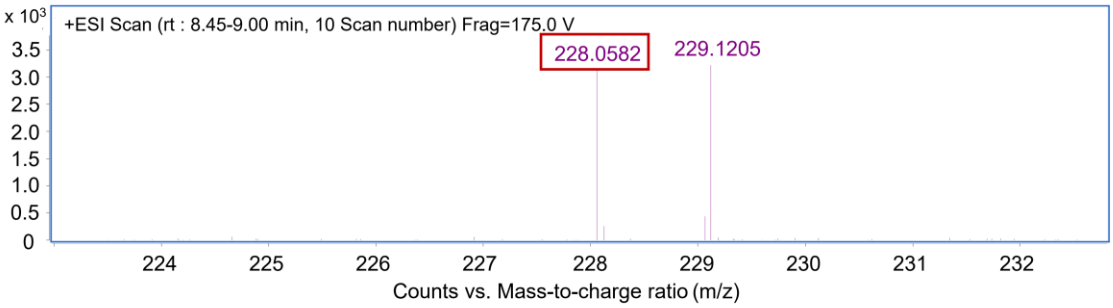
+ESI Scan (rt : 8.45-9.00 min, 10 Scan number) Frag=175.0 V
228.0582
229.1205
Counts vs. Mass-to-charge ratio (m/z)

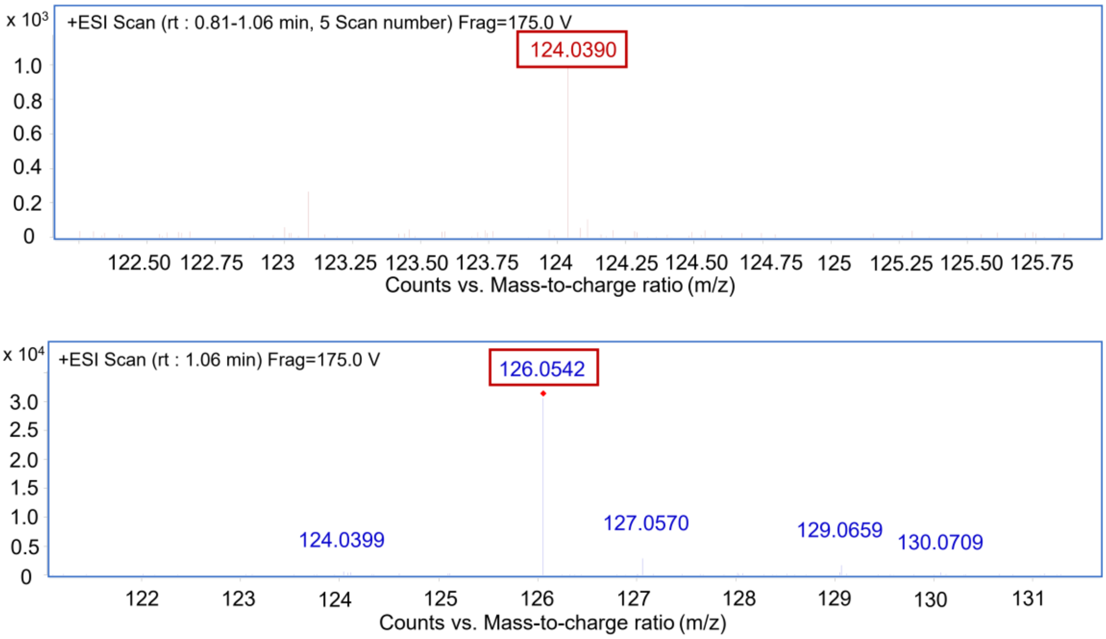

Supplementary Figure 49. Raw data of LCMS. (Liquid chromatography: Agilent 1290UPLC, Mass spectrometry: Agilent QTOF6550, mobile phase A: 0.1 % formic acid aqueous solution; Mobile phase B: acetonitrile solution; Flow rate: 0.3 ml/min, sample size: 5 ul, waters BEH C18 column, MS scanning range: primary 50-1000 m/z, ESI+ mode: voltage 4000 V, ESI- mode: voltage 3200 V.).

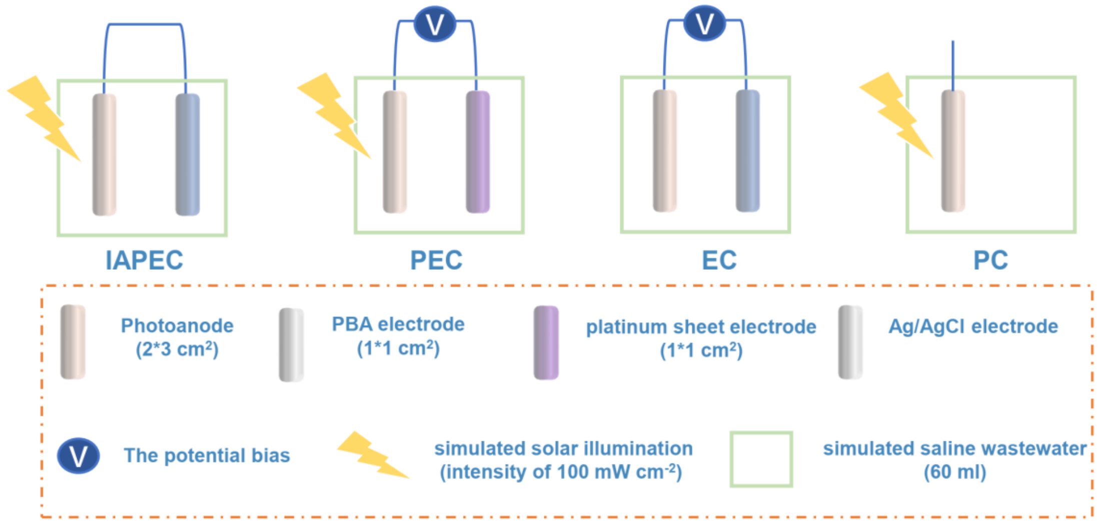

Supplementary Figure 50. A schematic diagram for the experimental conditions of IAPEC, PC, EC and PEC.

Supplementary Table 1. Fe pre-edge peak analysis on ex situ samples and the average oxidation state of Fe species in samples.

Sample	pre-edge peak energy (eV)	Edge energy (eV)	Average oxidation state
Fe foil	-	7112	+0
FeO	7112.43	7123	+2
Fe_2O_3	7115.07	7128.1	+3
Before Na^+ ions insertion	7114.93	7127.6	+2.9
After Na^+ ions insertion	7112.27	7123.4	+2.1

E_0 values obtained from the first derivation spectral，which is linearly related to the Fe valence state.

Supplementary Table 2. EXAFS fitting parameters at the Fe K-edge for various samples.

Sample	Shell	CN^a	$R(\text{Å})^b$	$\sigma^2(\text{Å}^2)^c$	$\Delta E_0(\text{eV})^d$	R factor
Fe K-edge ($S0^2$ = 0.811)						
Fe foil	Fe-Fe	8*	2.474±0.003	0.0052	7.1	0.0020
	Fe-Fe	6*	2.854±0.005	0.0067		
Before Na insertion	Fe-C	5.9±0.3	2.006±0.026	0.0089	2.7	0.0030
	Fe-C-N	2.8±0.5	3.014±0.025	0.0078	12.4	
	Fe-M	1.5±0.6	3.273±0.023	0.0111	-5.7	
After Na insertion	Fe-C	6.1±0.2	2.004±0.007	0.0055	0.9	0.0055
	Fe-C-N	3.3±0.5	2.917±0.011	0.0084	-1.3	

[a]*CN*, coordination number; [b]*R*, the distance to the neighboring atom; [c]σ^2, the Mean Square Relative Displacement (MSRD); [d]ΔE_0, inner potential correction; *R* factor indicates the goodness of the fit. $S0^2$ was fixed to 0.811, according to the experimental EXAFS fit of Fe foil by fixing CN as the known crystallographic value. * This value was fixed during EXAFS fitting, based on the known structure of Fe. Fitting range: $3.0 \le k$ (/Å) ≤ 14.2 and $1.0 \le R$ (Å) ≤ 3.0 (Fe foil); $3.0 \le k$ (/Å) ≤ 10.5 and $1.0 \le R$ (Å) ≤ 4.0 (1); $3.0 \le k$ (/Å) ≤ 12.7 and $1.0 \le R$ (Å) ≤ 3.0 (2); $3.0 \le k$ (/Å) ≤ 13.9 and $1.0 \le R$ (Å) ≤ 3.5 (3). A reasonable range of EXAFS fitting parameters: $0.700 < S_0^2 < 1.000$; $CN > 0$; $\sigma^2 > 0$ Å^2; $|\Delta E_0| < 15$ eV; R factor < 0.02.

Supplementary Table 3. Comparison of the photoelectrochemical performance of various PEC-Cl.

References	Condition	Photoelectrode	Degradation Rate	Chlorate
Environ. Pollut. 2020, 267 115605	50 mM NaCl, 350 W Xe lamp, 1.36 V vs. RHE, pH=7	$WO_3/BiVO_4$	30 ppm urea 97 %, 90 min	46.3 mg L^{-1}
Environ. Sci. Technol. 2019, 53, 6945−6953	50 mM NaCl, 150 W xenon lamp, 1.7 V vs. Ag/AgCl, pH= 5	Sb−SnO_2/WO_3 (16 cm^2)	30 ppm ammonia-N 99.2 %, 90 min	26.7 mg L^{-1}
Appl. Catal. B: Environ. 2021, 296, 120387	0.1 M NaCl, 300 W Xe lamp, AM 1.5, 1.2 V vs. RHE, pH = 3	TiO_2	10ppm phenol, 99.9 %, 120min	2.3 mg L^{-1}
J. Hazard. Mater. 2021, 402, 123725	300 mg L^{-1} NaCl, 350 W Xe lamp, pH = 9	$WO_3/BiVO_4$ (2.5*5 cm^2)	10 ppm ammonia-N, 99.3 %, 120 min	18.3 mg L^{-1}
Environ. Sci. Technol. 2019, 53, 9926−9936	50 mM NaCl, 300-W Xe arc lamp, 0.5 V vs. Ag/AgCl, pH = 4	WO_3 film (1*2 cm^2)	50 μM 4-CP, 99.9% 30 min	150.3 mg L^{-1}
J. Hazard. Mater. 2023, 443, 130363	50mM NaCl, 300 W Xe lamp, AM 1.5, 0.5 V vs. SCE, pH = 4	O_v-TiO_2 (2*2 cm^2)	20 ppm 4-CP, 99%, 45min	1.0 mg L^{-1}
This work (IAPEC system)	50 mM NaCl, 300 W Xe lamp, AM 1.5, pH = 6	TiO_2 (4.91 cm^2)	20 ppm MB 99%, 15 min	0.55 mg L^{-1}

Supplementary Table 4. The pH changes of simulated saline wastewater before and after degradation with different initial pH values.

pH value	2	4	6	8	10
Before degradation	2.13	4.06	6.21	8.15	10.01
After degradation	1.97	2.45	2.46	2.44	2.54

Supplementary Table 5. Hirshfeld charges and calculated f^-, f^+, f^0 and CDD of $C_{12}H_{10}N_3S^+$.

Atom	q(N)	q(N+1)	q(N-1)	f^-	f^+	f^0	CDD
1(C)	0.0989	0.0485	0.1411	0.0422	0.0504	0.0463	0.0082
2(C)	-0.058	-0.0874	-0.0074	0.0506	0.0294	0.04	-0.0212
3(C)	0.0072	-0.032	0.0261	0.0189	0.0392	0.0291	0.0202
4(C)	0.0377	-0.0013	0.1019	0.0642	0.039	0.0516	-0.0252
5(C)	-0.0151	-0.0613	0.0137	0.0289	0.0462	0.0375	0.0173
6(C)	-0.0336	-0.0731	0.0189	0.0525	0.0395	0.046	-0.013
7(C)	0.0378	-0.0014	0.1021	0.0643	0.0392	0.0517	-0.0251
8(C)	0.0072	-0.032	0.0262	0.019	0.0392	0.0291	0.0203
9(C)	-0.058	-0.0874	-0.0073	0.0507	0.0294	0.04	-0.0213
10(H)	0.0681	0.0501	0.0891	0.021	0.018	0.0195	-0.003
11(C)	0.099	0.0485	0.1413	0.0423	0.0505	0.0464	0.0082
12(C)	-0.0334	-0.073	0.0191	0.0525	0.0396	0.0461	-0.013
13(C)	-0.0151	-0.0614	0.0137	0.0289	0.0462	0.0376	0.0173
14(H)	0.0681	0.0501	0.0892	0.021	0.018	0.0195	-0.003
15(H)	0.0636	0.0401	0.0806	0.017	0.0236	0.0203	0.0066
16(H)	0.0703	0.0511	0.0918	0.0215	0.0192	0.0204	-0.0023
17(H)	0.0703	0.0511	0.0918	0.0215	0.0192	0.0204	-0.0023
18(H)	0.0636	0.0401	0.0806	0.017	0.0235	0.0203	0.0066
19(N)	-0.1235	-0.2231	-0.0987	0.0248	0.0997	0.0622	0.0749
20(S)	0.13	0.0262	0.17	0.0399	0.1038	0.0719	0.0639
21(N)	-0.0778	-0.1296	0.0122	0.09	0.0517	0.0709	-0.0383
22(H)	0.1674	0.1465	0.1977	0.0303	0.0209	0.0256	-0.0095
23(H)	0.1679	0.1469	0.1982	0.0304	0.021	0.0257	-0.0094
24(N)	-0.0779	-0.1296	0.0119	0.0898	0.0517	0.0708	-0.0382
25(H)	0.1678	0.1469	0.1982	0.0304	0.021	0.0257	-0.0094
26(H)	0.1674	0.1466	0.1977	0.0303	0.0208	0.0256	-0.0095

Supplementary Table 6. Hirshfeld charges and calculated f^-, f^+, f^0 and CDD of $C_6H_6NO_2^+$.

Atom	q(N)	q(N+1)	q(N-1)	f^-	f^+	f^0	CDD
1(C)	0.1162	0.066	0.1938	0.0776	0.0501	0.0639	-0.0275
2(C)	-0.0923	-0.1303	0.1161	0.2085	0.038	0.1232	-0.1705
3(C)	0.115	0.0571	0.1937	0.0787	0.058	0.0683	-0.0207
4(C)	0.2458	0.0912	0.2689	0.023	0.1546	0.0888	0.1316
5(C)	0.0097	-0.0557	0.0566	0.0469	0.0654	0.0562	0.0185
6(C)	0.0944	-0.0371	0.1166	0.0222	0.1315	0.0768	0.1093
7(H)	0.0659	0.0396	0.1293	0.0634	0.0264	0.0449	-0.037
8(H)	0.1028	0.0688	0.1243	0.0215	0.034	0.0278	0.0125
9(H)	0.1085	0.0606	0.1268	0.0183	0.0479	0.0331	0.0296
10(O)	-0.029	-0.1507	0.0019	0.0309	0.1217	0.0763	0.0908
11(H)	0.2737	0.2311	0.2871	0.0134	0.0426	0.028	0.0292
12(O)	-0.3487	-0.4554	-0.1686	0.18	0.1068	0.1434	-0.0732
13(N)	-0.0316	-0.0995	0.0961	0.1277	0.0679	0.0978	-0.0597
14(H)	0.1857	0.1573	0.2285	0.0428	0.0284	0.0356	-0.0144
15(H)	0.1838	0.1572	0.2289	0.0451	0.0266	0.0358	-0.0185

Supplementary References

1. Yan, F. et al. Efficient urine removal, simultaneous elimination of emerging contaminants, and control of toxic chlorate in a photoelectrocatalytic-chlorine system. *Environ. Pollut.* **267**, 115605 (2020).
2. Zhang, Y. et al. Extremely efficient decomposition of ammonia N to N_2 Using ClO• from reactions of HO• and HOCl generated in situ on a novel bifacial photoelectroanode. *Environ. Sci. Technol.*, **53**, 6945–6953 (2019).
3. Li, X. et al. The ClO• generation and chlorate suppression in photoelectrochemical reactive chlorine species systems on $BiVO_4$ photoanodes. *Appl. Catal. B: Environ*. **296**, 120387(2021).
4. Zhang, Y. et al., Efficient ammonia removal and toxic chlorate control by using $BiVO_4/WO_3$ heterojunction photoanode in a self-driven PEC-chlorine system *J. Hazard. Mater.* **402**, 123725(2021).
5. Koo, MS. et al. In Situ Photoelectrochemical Chloride Activation Using a WO_3 Electrode for Oxidative Treatment with Simultaneous H_2 Evolution under Visible Light. *Environ. Sci. Technol*. **53**, 9926-9936 (2019).
6. Wu, JB. et al. activation of chloride by oxygen vacancies-enriched TiO_2 photoanode for efficient photoelectrochemical treatment of persistent organic pollutants and simultaneous H_2 generation. *J. Hazard. Mater.* **443**, 130363(2023).
